# Supplementary material for: Digitized endocasts and brains: a perspective on measurements and historical analyses of the evolution of 172 fossil and extant amniote specimens
Source: PeerJ. 2025 Sep 16;13:e19826. doi: 10.7717/peerj.19826 (PMC12447954; doi:10.7717/peerj.19826)
Supplement: Supplemental Information 1 [file peerj-13-19826-s001.docx]

**Supplementary Results**

Digitized Endocasts and Brains: Measurements and Analyses of the Evolution of 172 Fossil and Extant Vertebrate Specimens

## Edinger’s Early Horses

### Body size estimates

*Evolution of the Horse Brain* (Edinger 1948) was my introduction to paleoneurology (see Jerison, 2001a), and I use it to introduce these results. Edinger’s photographs and drawings of horse species (Edinger 1948) have frequently been used to illustrate progressive brain evolution (MacFadden, 1992; Savage & Long, 1986; Simpson, 1951). **Fig. 2** shows scans of endocasts of five of Edinger’s species and adds Radinsky’s (1976b) *Hyracotherium* (AMNH FM 55268 = FMNH PM 59207); Edinger’s “*Eohippus*” (YPM 11694). The taxonomy of the American *Hyracotherium* is uncertain because the generic name was only correctly applied to the European species (Hooker, 1994). Secord et al. (2012) offered *“Sifrhippus”* as its new generic name; the species *tapirinum* remains its present identification at AMNH; here, the Radinsky terminology in used.

The digitized models of the bodies presented in **Figs. 3 and 4** are scans of Gidley’s (1927) careful sculptures, which determined the length, surface area, and volume. To enable the data in **Fig. 2** to be used to analyze *Hyracotherium*, the mounted skeleton was measured and reported a head length of 137 mm and body length of 559 mm, a 69.6 cm head+body length (courtesy of Dr. John Harris, then Chief Curator of the George C. Page Museum (LACM)). The volume and length of the model were scaled up (volume = 90.8 ml, length = 14.2 cm) and the model’s volume was multiplied by the cube of the ratio of the lengths (69.6 cm/14.2cm)^3^ to estimate a body size of 10,692 ml (~10.7 kg), which compares to an estimated body size of 9 kg (Radinsky, 1976b) using a regression analysis of the skull size (Jerison, 1973). Similarly, the AMNH mounted specimen of *Mesohippus bairdi* was photographed and measured (Dr. Ted Macrini, formerly at AMNH (**Fig. 3D**)) and the Smithsonian mounted specimen, USNM 244183, was measured (Dr. Robert Purdy, USNM). USNM 244183 and AMNH 1477 had almost identical body lengths (108 cm vs. 109.2 cm). Therefore, the body weight for *Mesohippus* was estimated to be 28.5 kg, which compares to MacFadden’s (1986) estimates of between 40 and 60 kg, which appeared to be high compared to the body-length ranges of living ungulates (Nowak, 1999).

Radinsky (1976b) judged Edinger’s “*Eohippus*”, found as a natural isolated endocast, to be a condylarth rather than an equoid, and it remains taxonomically unidentified.

### Neocorticalization and encephalization

The endocast measurements and neocorticalization of Edinger’s equoids, a zebra, and two domesticated horses (a pony and a draft horse, reflecting body size variations within the domesticated species) according to geological age are presented in **Table S1** and **Fig. S1**.

#### Table S1. Measurements of the endocasts of 12 of Edinger’s equoids.

| **Species** | **E** (ml) | **S** (cm^2^) | **NC** (cm^2^) | **P** (ml) | **MYA** |
| --- | --- | --- | --- | --- | --- |
| 0. "Tillyhorse" YPM VP 11694 | 13.97 | 38.74 | 7.12 | n/a | 52.9 |
| 1. *Hyracotherium* AMNH FM 55268 | 24.162 | 57.74 | 10.66 | 10700 | 52.9 |
| 2*. Mesohippus AMNH FM 9814* | 86.422 | 130.1 | 40.8 | 28500 | 32 |
| 3*. Merychippus* AMNH FM 71150 | 231.794 | 229.9 | 40.8 | 110000 | 15 |
| 4*. Pseudhipparion* AMNH FM 70025 | 168.437 | 1941.2 | 92.7 | 50000 | 13.6 |
| 5*. Pliohippus* FMNH P 15870 | 288.762 | 270.2 | 134.6 | 169700 | 12 |
| 6*. Neohipparion* P15871 | 227.465 | 224.8 | 107.7 | 172100 | 12 |
| 7*. Cormohipparion* PM59220 | 363.956 | 309.3 | 175.7 | 151000 | 11 |
| 8*. Equus LACMHC 3500-17* | 868.78 | 541.3 | 317.6 | 550000 | 0.5 |
| 9*. Equus sp.* (Zebra) LACM Mammals 342 | 625.423 | 431.5 | 204.3 | 30000 | 0 |
| *Equus caballus* (Arabian) LACM | 669 | 444.4 | 232.7 | 400000 | 0 |
| *Equus caballus* (Draft Horse) LACM | 881 | 540.1 | 273.2 | 800000 | 0 |

E: specimen volume; S: surface area; NC: neocortex; P: body size; MYA: millions of years ago.

The Arabian and draft horse can be represented in Fig. 12 by Species 8 and 9.

####
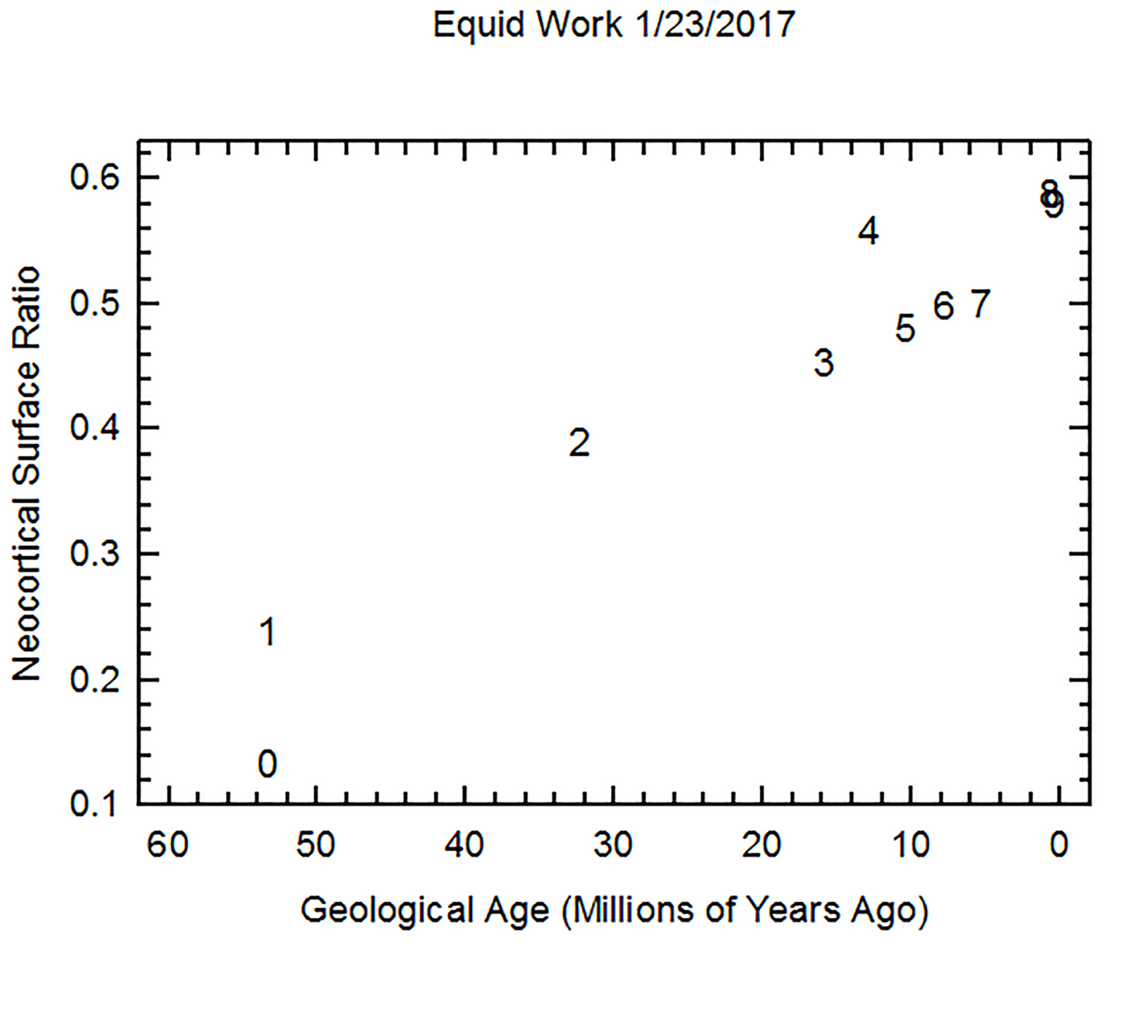


#### Figure S1. Neocorticalization in equoids. Neocorticalization in Edinger’s horses with the regression line determined on a smaller sample of fossils. Data relabeled in Table S1 with Edinger’s “*Eohippus*”, my Tillyhorse (Point 0), and Radinsky’s *Hyracotherium* (Point 1).

Like Edinger, Radinsky (1976b) discussed the presence of visible cortical sulci in *Hyracotherium*, which is likely to be a correlate of brain size (24.2 ml vs. 14 ml) and comparable to brains in this size range in living species. More unusual was the size of the *Hyracotherium* cerebellum, which was larger relative to the whole endocast than would be expected according to the quantitative analysis of living brains (Stephan, Frahm & Baron, 1981; Jerison 1991). It is an interesting exception to the uniformitarian hypothesis and is explained by the small forebrain in early mammals. The cerebellum is probably a correct size for its neural control functions, but its ratio to the whole brain in this early Eocene equoid is high because early forebrains were relatively small.

The endocast of *Mesohippus* (**Fig. 4A**) was larger, more encephalized, and much more convoluted than that of *Hyracotherium* (**Fig. 3A**). Except at its anterior border, the rhinal fissure is a dark line in **Fig. 3A** with the forebrain surface area dorsal to this line being neocortical. Neocorticalization in *Mesohippus* is 38% (Point 2 in **Fig. S1**).

With the exception of the smaller *Pseudhipparion* (renamed from *“Griphippus”* in the FMNH catalogue), the Neogene equids are in the body size range of living equids, and MacFadden’s regression estimates of body size for the larger species are acceptable. Gidley (1927) prepared careful models of several of these (*Merychippus*, *Neohipparion*, and *Equus*), but these could not be digitized in order to measure their body sizes. *P. (Griphippus) gratum* FMNH PM 59211 was a small Miocene equid with an estimated body weight of 50 kg (pers. comm. and Hulbert, 1993). For *Merychippus* and larger fossils, rounded averages from MacFadden’s (1986) regressions, similar to previously published (Jerison, 1973), have been used.

**Fig. 5** shows the remainder of the endocasts of Edinger’s equoid genera: three fossil genera and three recent genera, including a zebra and two domesticated horses (a pony and a draft horse, reflecting body size variations within the domesticated species). The encephalization of the equoid sample is plotted in **Fig. S2** using endocast sizes and body weights, with two living domesticated equids removed for clarity (centered around Point 9). The statistically determined regression equation at slope 3/4 is similar to my preferred theoretical allometric “regression” at slope 2/3.


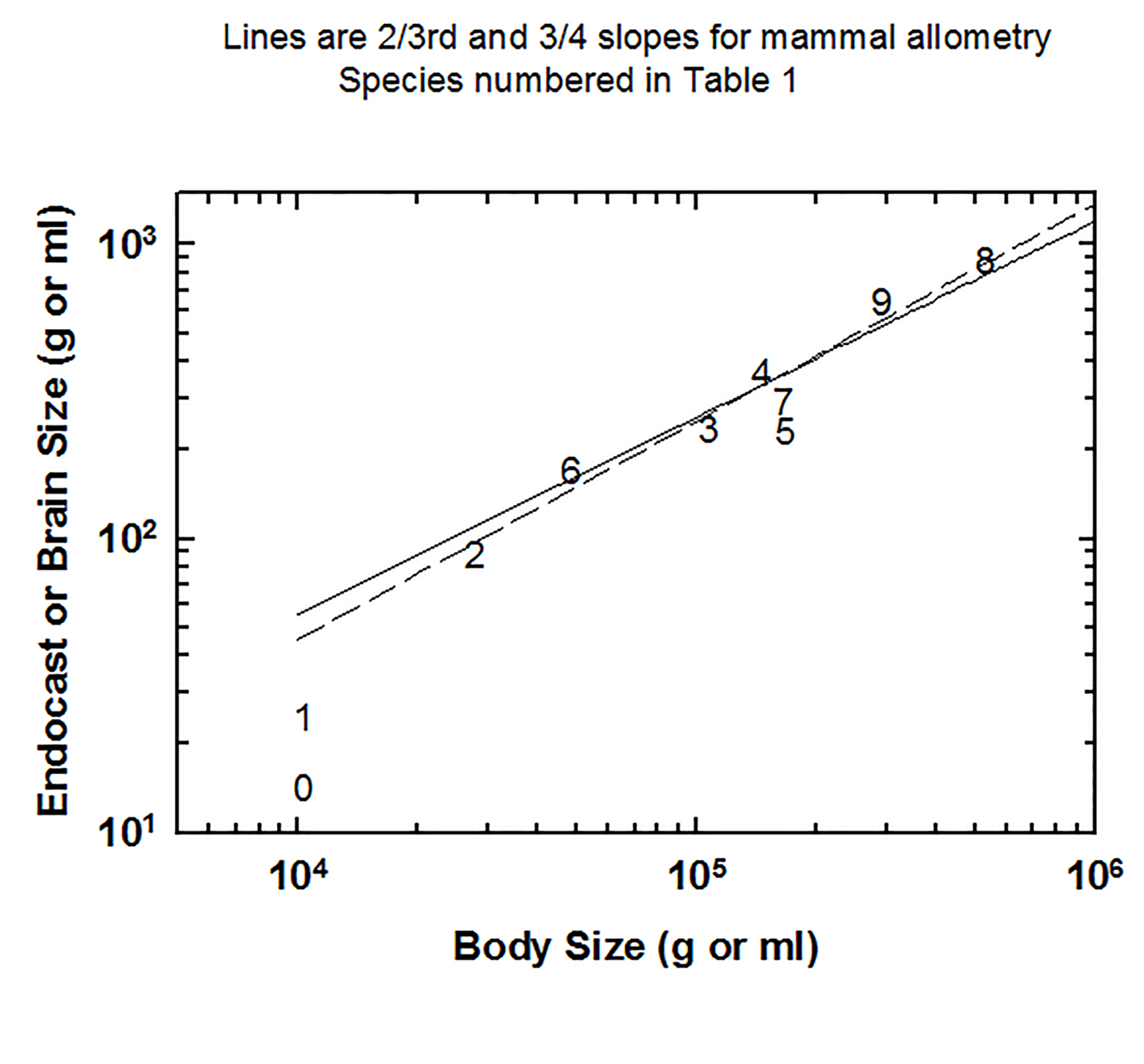


#### Fig. S2. Encephalization in equoids. Encephalization in Edinger’s horses numbered as in Table S1. Regression lines are of living mammals within the equoid body size range for log-log data. The least squares regression equation as a power function for living mammals (Martin, 1990) is *Y* = 0.05 *X* ^0.74^, and the “regression” through the mammalian centroid (Jerison, 1973, 2001a) is *Y* = 0.12 *X* ^2/3^. See also Fig. S1.

Given that encephalization in the early Eocene was at the grade of the equoid *Hyracotherium* and comparable to its Eocene contemporaries, it was below the encephalization relative to the allometry of later equids. With the appearance of *Mesohippus* in the late Eocene or early Oligocene at about 32 Ma (Species 2 in **Fig. S2**), encephalization among equids reached the average grade of living mammals. The later equids, such as the La Brea horse (**Fig. 5** and Point 8 in **Fig. S2**) and the living horses and zebras are all at about the present average mammalian grade of encephalization.

Overall, the evolution of encephalization in many groups appears to have followed a relatively early increase to a particular grade followed by stasis at that grade. *Hyracotherium* was about half as encephalized as average extant mammals. *Mesohippus,* at a body size *P* = 28.5 kg and endocast volume *E* = 86 ml, was within the average range for living mammals. In living equids, the range is 0.75 < *EQ* < 1.11 (cf. Count, 1947). In the Oligocene to Pleistocene equids, the range is 0.63 < *EQ* < 1.12.

Reliable body size data for the early Eocene condylarth that Edinger called *“Eohippus”* is not available, but I previously estimated it as 13 kg (Jerison, 1973) and here modify it to 10.7 kg, the same size as *Hyracotherium* in **Table S1** and **Fig. S2**, due to a reduction in endocast volume from a previous measurement of 15 g the current 3D measurement of 14 g. These estimates lead to *EQ* = 0.24, a typical value for late Paleocene and early Eocene mammals, one-quarter to one-third as encephalized as extant species of similar body weights. It is worth adding that many living insectivores and didelphids have a similar low grade of encephalization; there are clearly many niches in which that grade of encephalization works.

# Description of all the endocasts

### Paleocene Fossils

*Titanoides*

The endocast of FMNH PM 8655 (**Fig. 6A**), probably *Titanoides primaevus*, was donated by Dr. William D. Turnbull to the FMNH collection in 1962. He collected it in the Tongue River Formation, Mercer County, Garrison Reservoir area, 2-3 miles north of Riverdale in North Dakota. According to dating of the area (Kihm & Hartman, 2004; Secord et al., 2006) *T. primaevus* lived 59.2 Ma. McKenna & Bell (1997) list this pantodont in the order Cimolesta, family Titanoideidae. From Savage & Long (1986), its body length was 160 cm and, from Eq. 2, this results in a body weight (heavy habitus) of 172 kg in the allometric analysis of encephalization. With respect to its endocast (**Fig. 6**), the length = 11.7 cm; *E* = 88.35 ml; *EQ* = 0.24; endocast surface area = 152.94 cm^2^; total neocortical surface area (twice the marked surface) = 18.98 cm^2^; and olfactory bulb surface area = 17.9 cm^2^. The neocorticalization ratio is 14.1%.

*Arctocyon*

On my first field trip to Europe in Paris at MNHN in 1964, I especially enjoyed working with this specimen of *Arctocyon primaevus*. Dr. Donald Russell showed me its skull, in which an endocast could be prepared by pouring molding compound into the cleaned cranial cavity. I already had a copy of an endocast that had been described in 1870 by Gervais, and I had recognized that an error must have been made in its preparation. Edinger (1964) illustrated and discussed the specimen, noting that Gervais had actually prepared a half endocast and combined it with a mirror image. I am uncertain how Gervais managed this, but **Fig. S3** shows my digital approach with a wombat skull, scanning the cranial cavity and inverting its digitized image.


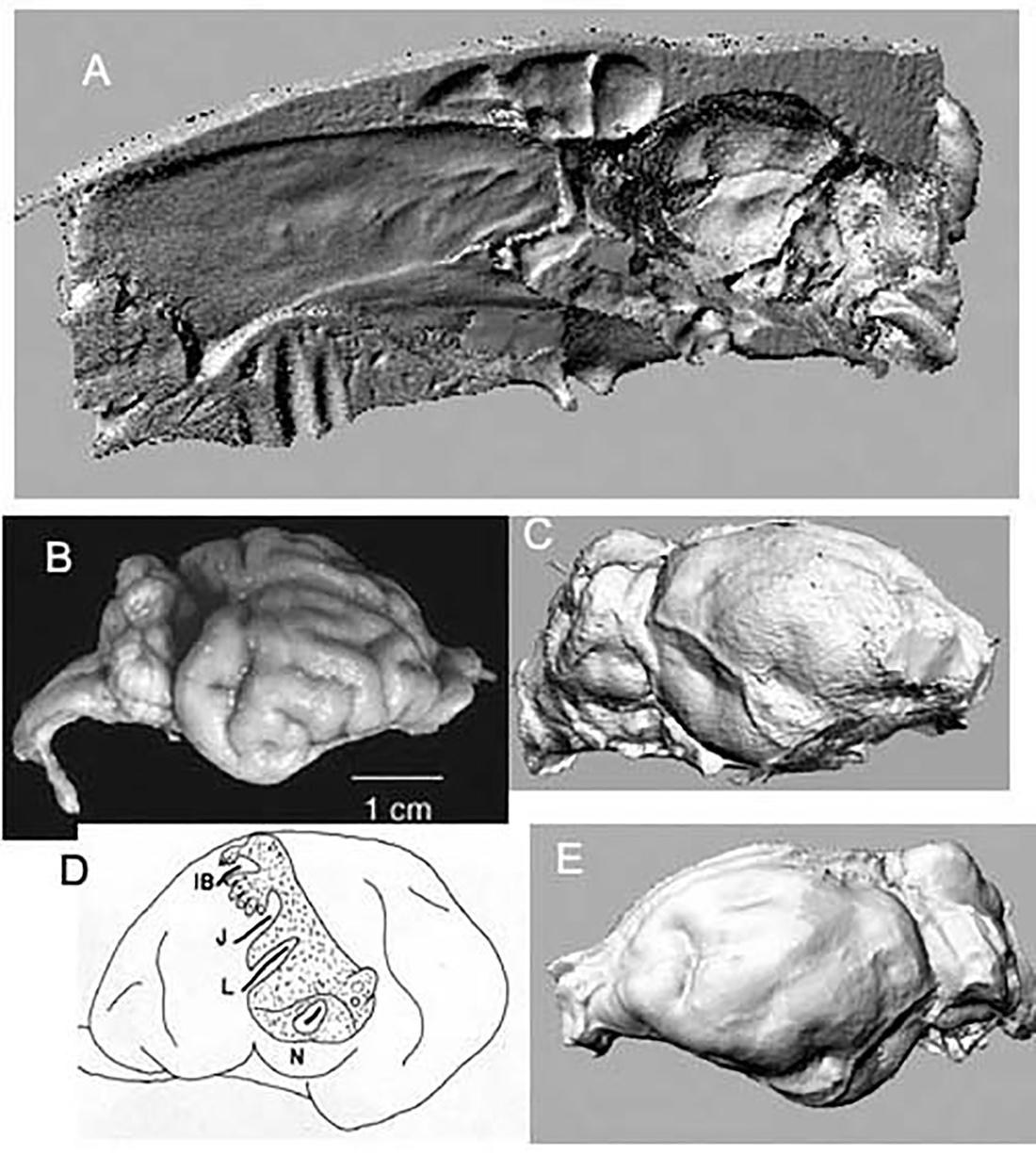


#### Fig. S3. Wombat brain and endocast. *Vombatus ursinus* (wombat) skull, brain, and endocast. (A) Skull (NMV Specimen C7780), anterior left. (B) Brain (WISC Specimen 64-11), anterior right, 55.4 g. (C) Endocast, anterior right, 73.7 ml. (D) Brain map, anterior left, showing somatosensory cortex as determined by Johnson (1980) from evoked potentials. (E) Silicone endocast, anterior left. Images of skull and endocasts prepared by the author.

While Edinger emphasized an error in the “cerebellum”, which lacked a vermis, I was even more impressed by the odd triangular shape of Gervais’s endocast. When we saw the new *Arctocyon* skull, I convinced Dr. Russell to allow me to prepare a new endocast from it. Sadly, my procedure involved a quick-fixing poisonous latex-like compound with which I worked at the time. It produced an excellent endocast, clearly different from Gervais’s, but I could not remove my entire mold successfully. Drs. Russell and Sigogneau were able to repair my damage and prepare a proper endocast, publishing it the following year (Russell & Sigogneau, 1965).

The scans in **Fig. 6B** are dorsal and lateral views of their endocast of *Arctocyon primaevus* (MNHN F CR700). The *Arctocyon* specimen is from the late Paleocene in the Cernay-Berru (Marne) in France. Dr. Thierry Smith (pers.comm.) was studying the region and wrote to me that the area is “late Thanetian” and that my estimated age of 58 Ma was reasonable. Its endocast volume is *E* = 7.1 ml, with the length (top of olfactory bulb to bottom of medulla) = 4.9 cm. The endocast volume of 38 ml and body weight of 86 kg, published in Jerison (1973), are typographical errors. The species body length is estimated as 87 cm (Argot, 2013), leading to an estimated body weight of 16 kg (*EQ* = 0.09). When I examined the fossil it was a creodont, but it was later reclassified a condylarth and it is now classified in the archaic order Procreodi. Working on the present study, my small Paleocene procreodont forces me to modify some long-held ideas, as discussed in the main text. The neocortical measurements of *Arctocyon* are presented in **Table 1**.

*Barylambda*

One specimen is listed as FMNH P 15573 and is shown on the right in **Fig. 6C**. The second specimen is listed as FMNH P 26075 and is shown on the left in **Fig. 6C**. Both specimens are now called *Leptolambda schmidti*. McKenna & Bell (1997) treated *Leptolambda* as a junior synonym for *Barylambda*, which is used here. These are the heaviest Paleocene pantodonts in my sample, order Cimolesta. At body lengths of just over 230 cm and heavy habitus, I estimated both of their weights to be 620 kg. Both specimens are from the DeBeque Formation in Colorado and dated as 56 Ma (**Table 1**). On specimen FMNH P 15573, I removed casts of cranial nerve roots by dissecting the digital image before determining the endocast volume to be 85.22 ml; surface area = 126.79 cm^2^; olfactory bulbs absent; neocortical surface area = 9.71 cm^2^; neocorticalization = 7.7%; and *EQ* = 0.10. Evidence of neocortex on this endocast is marginal: two small anterior mounds on the frontal surface and a poorly marked olfactory tract.

The forebrain of specimen FMNH P 26075 (**Fig. 6C**, left) is more brain-like. Expanded cerebellar representation in this specimen probably reflects fluid surrounding the cerebellum. Unlike the *Arctocyon* endocasts shown in **Fig. 6B**, it is not divided into vermis and lobules. The volume of the endocast of specimen P 26075 is *E* = 98.13 ml; endocast length (olfactory bulbs to medulla) = 8.7cm; total surface area = 143.30 cm^2^; olfactory bulb surface area = 17.77 cm^2^; remainder surface area = 125.52 cm^2^; neocortex = 6.46 cm^2^; and neocorticalization = 5.15%. The rhinal fissure is only marginally recognizable. These two *Barylambda* endocasts are among the least brain-like of the current specimen set, with both showing much-enlarged “medulla” in the posterior area, definite non-neural space in the foramen magnum for blood vessels, meninges, and sinuses.

*Phenacodus*

The endocast of the condylarth *Phenacodus primaevus* (AMNH FM 4369 = FMNH PM 59042) is the specimen described in Cope’s “Bible” (Cope, 1883, pp. 435-442). It is from the Big Horn Basin of Northern Wyoming, Wind River Region. It can be dated as Biochron zone Wa1 or Wa2, about 54 Ma (Dr. Gregg Gunnell, pers. comm.). The temporal range of the species is from late Paleocene to middle Eocene, and its endocast is also representative of the late Paleocene. The endocast was originally prepared by Cope or his staff in the 1870s and is shown in **Fig. 6D**. The cast itself was unusually smooth for a cast prepared from a fossil skull compared, for example, with the equally complete endocast of *Arctocyon.* It may have been smoothed to look more brain-like when Cope prepared it. The *Phenacodus* head+body was about 150 cm long; body weight *P* = 82 kg; *E* = 30.82 ml; *EQ* = 0.14; endocast length (olfactory bulbs to medulla) = 7.7 cm; surface area = 72.75 cm^2^; olfactory bulbs = 10.33 cm^2^; remainder area = 62.42 cm^2^; neocortex = 10.01 cm^2^; and neocorticalization = 16%.

### Early Eocene Fossils

The Early Eocene dates in this report are from 54 Ma to 42 Ma.

*Coryphodon*

When I first saw the endocast of *Coryphodon hamatus* (YPM VP 11331 = FMNH PM 59241), its cerebellum struck me as not at all brain-like. The specimen came from the archaic Cimolesta located in the Wyoming Uinta County Evanston and dated to about 52 Ma (Woodburne, 2004). It was presumably prepared at the Yale Peabody Museum under O. C. Marsh’s supervision over a century ago. That endocast alerted me to the occasional large errors in preparation. I thought at the time that my observation was original but then discovered that Edinger (1933) had also noted the likely error. Evidently, fossil petrosal bone had been drilled away, leaving massive lateral bubbles that enlarged the “cerebellum” cast. With the digitized image, I simply deleted the bubbles and measured the remaining endocast (**Fig. 7A**), which produced sufficient correction even in the absence of more cerebellar-like edges. *Coryphodon* was about 2 meters long with a heavy habitus, indicating a body weight of about 400 kg. The vertical length of *Coryphodon* endocast = 11.8 cm; endocast volume *E* = 90.6 ml; *EQ* = 0.14; endocast surface area = 140.94 cm^2^; olfactory bulbs = 13.66 cm^2^; remainder area = 127.28 cm^2^; neocortex = 24.34 cm^2^; and neocorticalization = 19.12%.

*Palaeosyops*

The endocast FMNH PM 59198 is probably *Palaeosyops leidyi*, a large brontothere (Perissodactyla), late Wasatchian, beginning of Bridgerian, and dated as 51.7 Ma for the neocorticalization analysis (**Fig. 7B**). From Osborn (1929), the body length is estimated as 198 cm, and from the appearance of the limbs in the mounted skeleton at AMNH, its habitus was normal (not heavy). This leads to a body weight estimate of 191 kg for allometric brain-body analysis; endocast vertical length = 14.2 cm; endocast surface area = 288.00 cm^2^; olfactory bulb area = 28.24 cm^2^; remainder = 259.76 cm^2^; neocortex = 40.20 cm^2^; and neocorticalization = 15.5%.

*Heptodon*

The genus of this early tapiroid is known to date to 55 Ma to 49 Ma. Without any knowledge about when or where my specimen was collected, I dated it as 52 Ma for the neocorticalization analysis. The animal was about 100 cm long with a body weight *P* = 24 kg. For the encephalization analysis, the endocast volume *E* = 43 ml and *EQ* = 0.43. The specimen is FMNH PM 59193, marked “MCZ” without further identification, and has length = 9.2 cm (**Fig. 7C**). Based on body weight, I prefer my Eq. 1 based on body length but note that the evidence of tooth size suggests a smaller, 15.5 kg body (Mendoza, Janis, & Palmqvist, 2006). Endocast surface area = 89.03 cm^2^; olfactory bulbs = 11.42 cm^2^; remainder = 77.6 cm^2^; neocortex = 20.96 cm^2^; neocorticalization = 27.0%. **Fig. 7C** shows a photograph of the endocast and another view of the scan to its right.

*Isectolophus*

*Isectolophus latidens* (FMNH PM 59179, copied from AMNH FM 12222) is a tapiroid from Bridger C-D and dated to 47 Ma (**Fig. 7D**). Its endocast volume *E* = 20 ml, including the olfactory bulb region preserved in its right hemisphere; length (olfactory bulb to medulla) = 6.6 cm. Holbrook, Lukas & Emry (2004) recorded its skull as 15.5 cm long and, assuming a body length of 78 cm, its body weight was about 12 kg, resulting in an *EQ* = 0.33. Endocast surface area of *Isectolophus* = 51.05 cm^2^; olfactory bulbs = 8.3 cm^2^; remainder = 42.75 cm^2^; neocortex = 9.03 cm^2^; and neocorticalization = 21.1%.

*Tillyhorse*

The identification of this endocast (YPM VP 11694; **Fig. 2**) as equoid was corrected by Radinsky (1976b). It was described by Edinger (1948) as *“Eohippus validus”* and continues to be described in some publications as an early equid brain. It is identified in the YPM database as “Condylarthra”. Edinger reported that it was from the upper Wasatchian, Almagré zone of Canyon Largo group, San Juan Basin, New Mexico. From Woodburne (2004), it appears to be Wa5 or Wa6, about 53 Ma. In Jerison (1973), it was described as *Hyracotherium*, with a 15 g endocast and a 13 kg body. While the brain size was a datum from the endocast, the body size was a complete guess. Nevertheless, neocorticalization could be determined because its total surface area, the surface area of its olfactory bulbs and the surface area of its neocortex, could be measured. Lacking an identification of its body, one cannot estimate body size for an allometric analysis of encephalization, although I graphed a new estimate of 10.7 kg (cf, Jerison, 1973). The endocast length = 6.4 cm, and neocorticalization data for the specimen were analyzed as follows: total endocast surface area = 49.52 cm^2^; olfactory bulb area = 4.83 cm^2^; remainder = 44.69 cm^2^; neocortex = 5.84 cm^2^; and neocorticalization = 13.07%.

*Hyracotherium*

This is the corrected “*Eohippus*” endocast that was prepared by Radinsky (1976b) from American *Hyracotherium* skulls in the Frick Collection (AMNH FM 55268 = FMNH PM 59207), where it is identified as *Hyracotherium tapirinum* (see also **Fig. 3**). The locality is Oak Creek, Castillo Pocket, Colorado, Horizon: Huerfano, and its geological age is 52.9 Ma. Its inclusion among the equids may be inappropriate, because *Hyracotherium* may be valid only for the European palaeothere species discussed by Owen (1841). Following a suggestion by Dr. Jerry Hooker (pers. comm. and Hooker, 1994) one can refer to it as an equoid, although the nomenclature is still debated. Its data are included in **Table S1** and **Table 1** and in the encephalization and neocorticalization plots in **Fig. S1 and S2**. *H. tapirinum* was somewhat larger than the skeleton at LACM. Its head+body length was about 88 cm (MacFadden, 1992, Fig. 12.9), leading to an estimated body size of 16 kg, one of the larger species of this early equoid. The endocast length (olfactory bulb to medulla) = 6.6 cm; volume *E* = 24.16 ml; surface area = 57.33 cm^2^; olfactory bulbs = 8.53 cm^2^; remainder = 48.8 cm^2^; neocortex = 10.66 cm^2^; and neocorticalization = 21.84%. I continue to use *P* = 10.7 kg, following my scale model and data derived from the LACM mounted skeleton.

*Meniscotherium*

Probably contemporaneous with and similar in brain size to the Tillyhorse, this specimen is *Meniscotherium robustum* (USNM V 19509), Early Wasatchian of Southern Wyoming. It has been described as about the size of a cocker spaniel, which on average weigh about 14 kg, but Gazin (1965) included pictures of the endocast and of a mounted *Meniscotherium* skeleton (not included here). Its head+body length was about 65 cm, leading to a body weight estimate of 6.5 kg, which is smaller than living adult cockers. This weight and a date of 53 Ma are used here. In a footnote to his review of *Hyracotherium,* Radinsky (1976b) suggested that the Tillyhorse was a specimen of *Meniscotherium,* which was common at that site. The narrow Tillyhorse forebrain proportions are significantly different, however, from the equally brain-like but differently proportioned *Meniscotherium* endocast illustrated by Gazin. Tillyhorse, therefore, remains an unidentified species, probably a condylarth. *Meniscotherium*, like *Hyracotherium*, was a relatively large-brained species for its time. Its 14.8 ml endocast indicates an *EQ* = 0.35. My endocast only had its dorsal surface, which made all measurements uncertain, in particular measurements of neocorticalization, although the dorsal half included the rhinal fissure.

*Hyrachyus*

Three Eocene specimens that I first saw listed as *Hyrachyus* were scanned. One was probably correctly described as *Hyrachyus modestus* (YPM VP 11082 = FMNH PM 59240), while a second had also been described as *Hyrachyus modestus* (YPM VPPU 10145) at YPM as well as *“Orthocynodon (Amynodon)”* at FMNH (FMNH PM 59177). The endocasts are similar but not identical. *Hyrachyus* were early rhinos or tapiroids. The second is described below as *Orthocynodon*, while the third specimen was much larger and discussed as *Amynodon*. The specimen numbers will keep them distinct, and I assume paleontologists expert in perissodactyl taxonomy, following Radinsky (1963, 1978), will review the specimens. I measured endocast volume of *H. modestus* (YPM VP 11082 = FMNH PM 59240) *E* = 69 ml and of *Orthocynodon* (YPM VPPU 10145 = FMNH PM 59177) *E* = 94 ml. My scan of the much larger *Amynodon* endocast (YPM VP 11453 = FMNH PM 59231) was *E* = 180.69 ml.

*Hyrachyus modestus* (YPM VP 11082 = FMNH PM 59240; **Fig. 8A**) is from the Wyoming Bridger Formation, and from Woodburne (2004) I estimated its age as 51.7 Ma. Savage & Long (1986) provided a head+body length as 1.6 meters, and my weight-length formula gives it a body weight *P* = 100 kg; *E* = 69 ml; and *EQ* = 0.27. I disagree with Radinsky’s (1978) estimates of head and body length = 122 cm and do not know why he chose *P* = 22 kg. Radinsky also reported *E* = 70 ml, presumably by using Archimedes’ method, which is close to my scan measurement. My endocast length for *H. modestus* is 8.7 cm. Surface area = 115.32 cm^2^; olfactory bulbs = 8.47 cm^2^; remainder = 106.85 cm^2^; neocortex = 29.75 cm^2^; and neocorticalization = 27.85%.

*Orthocynodon*

This specimen (YPM VPPU 10145 = FMNH PM 59177; **Fig 8B**) is still listed as *Hyrachyus modestus* in the YPM Catalogue, as it was reported by Radinsky (1963, 1978). It is from the Wyoming Bridger Formation, dated to 50 Ma. Radinsky (1978) had reported a body weight of about *P* = 50 kg, which was clearly too small; it was heavier than *H. modestus*, and I work with *P* = 150 kg. Endocast length = 9 cm; volume *E* = 94 ml; and *EQ* = 0.28, about the same as *H. modestus*. The surface area = 140.52 cm^2^; olfactory bulbs = 21.25 cm^2^; remainder = 119.27 cm^2^; neocortex = 30.82 cm^2^; and neocorticalization = 25.8%, again about the same as *H. modestus*.

*Amynodon*

According to Wall (1998), *Amynodon* ranged from 46.7 Ma to 39.5 Ma. I scanned what were originally listed as two specimens in the FMNH catalogue, FMNH PM 59231 and FMNH PM 59177. The first of these, copied by Radinsky from YPM VP 11453 and listed at FMNH by Radinsky as *A. erectus*, is listed by YPM as *A. advenus*. The second specimen was listed as *“Orthocynodon (Amynodon)”* (sic) and is YPM VPPU 10145 (= FMNH PM 59177), probably also prepared by Radinsky. His placing the species in parentheses must have reflected his uncertainty at that time about the name. The YPM describes it as *Hyrachyus modestus* in its catalogue, and Radinsky appears to have used the name. In view of these uncertainties, the specimen's measurements are listed under *Orthocynodon* above. With respect to the first specimen, the YPM catalogue lists YPM VP 11453 (= FMNH PM 59231) as *Amynodon advenus* Marsh from Uintah County, Utah, and this can be dated to about 46 Ma. The scans are shown in **Fig. 8C**. From Wall (1982, 1998), skull length = 45 cm; body length = 220 cm; *P* = 263 kg. For the encephalization analysis, its endocast volume *E* = 181 ml and *EQ* = 0.37. Endocast surface area = 239.39 cm^2^; olfactory bulbs = 12.41 cm^2^; remainder = 226.98 cm^2^; neocortex = 75.77 cm^2^; and neocorticalization = 33.4%.

*Eomoropus*

Scans of the endocast of this Eocene early chalicothere (Perissodactyla) are shown in **Fig. 8D**. I have seen only the skull and endocast. The skull was just under 30 cm long and suggested an animal about 120 cm in length; *P* = 40 kg. The endocast is of *Eomoropus amarorum* (FMNH PM 59182 copied from AMNH FM 5096); length = 6.8 cm; and volume *E* = 37 ml. It was recovered from the Washakie Formation with the suggested geological age of 44 Ma. The *EQ* = 0.26; endocast surface area = 80.41 cm^2^; olfactory bulbs = 1.76 cm^2^; remainder = 78.66 cm^2^; neocortex = 23.46 cm^2^; and neocorticalization = 29.8%.

*Mesatirhinus junius*

My two scanned specimens of *Mesatirhinus* are AMNH FM 1509 (= FMNH PM 59196) and YPM VPPU 10041 (= FMNH PM 59197), both from the Bridger Eocene, about 46.5 Ma. The first is probably *M. petersoni* (AMNH FM 1509; **Fig. S9A**), and the second (YPM VPPU 10041; **Fig. S9B**) is probably *Mesatirhinus junius*; both are now identified as *Mesatirhinus junius* (Mihlbachler, 2008). YPM VPPU 10041 came from Sweetwater County, Wyoming. Their data are presented in **Table 1.**

There are ambiguities in the history of the nomenclature. FMNH PM 59197 was copied from Marsh’s specimen YPM VPPU 10041. It is recorded in the Yale Peabody Museum catalogue as *M. junius*, family Brontotheriidae in Perissodactyla. According to the Yale catalogue, both specimens were probably collected from the Bridger Formation and the Washakie Formation and are dated to approximately 46 Ma. This *M. junius* may have been somewhat larger than *“M. petersoni*”, but I take them both as *P* = 350 kg. The Yale Peabody Museum also records it as *Tetheopsis ingens* and *Eobasileus commutus*; that is, as uintatheres, but I treat it as a brontothere, Perissodactyla. *Mesatirhinus* is thus “progressive” rather than “archaic” with respect to survival and extinction. It is shown in **Fig. S9A**; the lateral view on the right shows the rhinal fissure. The endocast length is 12.3 cm; *E* = 189.5 ml; *EQ* = 0.32; endocast surface area = 227.32 cm^2^; olfactory bulbs = 14.56 cm^2^; remainder = 212.76 cm^2^; neocortex = 58.23 cm^2^; and neocorticalization = 27.37%.

*Mesatirhinus petersoni*

My endocasts are shown in **Fig. S9B**. The endocast is of *M. petersoni* (AMNH FM 1509 = FMNH PM 59196). The length = 13.2 cm; *P* = 350 kg; *E* = 146.90 ml; *EQ* = 0.25; endocast surface area = 221.06 cm^2^; olfactory bulbs = 24.68 cm^2^; remainder = 196.38 cm^2^; neocortex = 37.17 cm^2^; and neocorticalization = 18.93%.

*Pachyaena and Mesonyx*

*Pachyaena ossifraga* (YPM VPPU 14708) is a mesonychid collected in 1947 from the Willwood Formation in Wyoming, dated to about 53 Ma. Its body size was estimated as about 65 kg (Zhou, Sanders, & Gingerich, 1992). Its endocast is illustrated in **Fig. S9C**. The endocast of *Mesonyx obtusidens* (FMNH PM 57139) was copied by Radinsky from YPM VP 13141 (Radinsky, 1976a). Only about half of the brain+matrix was present, and I removed excess matrix when preparing its digital image (**Fig. S9D**). A dramatic evolutionary development in the brain of mesonychids is evident in comparing the *M. obtusidens* endocast with the earlier mesonychid, *Pachyaena ossifraga* (Zhou et al., 1992). The *Pachyaena* endocast length = 9.2 cm, while the *Mesonyx* endocast length = 8.2 cm. Note the poorly defined olfactory tracts and bulbs in the older specimen and their complete absence in the later one.

*Mesonyx obtusidens* is from Bridger B in Wyoming, dated to about 48 Ma. Savage & Long (1986) illustrated the animal and estimated its body length as 150 cm, which resulted in a body weight *P* = 82 kg. Radinsky had estimated its body weight from the skull length as about 50 kg. Here I use the middle value of 65 kg, the same as the estimated body weight suggested by Zhou et al. (1992) for *P. ossifraga*. The endocast volume of the earlier mesonychid *P. ossifraga* is *E* = 32.66 ml; *EQ* = 0.17. The allometric expected brain size in a 65 kg living species is 194 ml, which would be the denominator of *EQ* in both mesonychids. My *Mesonyx obtusidens* fragment is 48 ml. Assuming that the volume of the fragment of *Mesonyx* is half the total volume (disregarding the absence of olfactory bulbs), the total volume *E* = 96 ml and *EQ* = 0.49, more than twice as encephalized as the older mesonychid.

For a context of *EQ*s in living carnivoran species weighing about 65 kg, here are some numbers from Van Dongen’s (1998) tables for living mammals. Zhou et al. measured the living hyena, *Hyaena crocutta*, for comparisons with *P. ossifraga*. The living hyena's reported body weight was 66 kg and its brain weight 161g; hence *EQ* = 0.82. The smaller living jaguar, *Panthera onca*, weighed 56 kg and had a 150 g brain, *EQ* = 0.85.

The forebrain and hindbrain in *Pachyaena ossifraga* are linearly aligned, similar to many Eocene species. The *M. obtusidens* endocast is bundled more spherically, comparable to that of *Eusmilus bidentatus* and *Felis catus* shown above. Its cerebellum, however, extends posteriorly beyond the forebrain. That my estimated total endocast was only half the volume of comparable living brains (*EQ* = 0.49) is of course part of the story of increasing encephalization in brain evolution.

The quantitative analysis of neocorticalization in the mesonychids is similar to their encephalization. The endocast surface area in *P. ossifraga* was 88.5 cm^2^, including olfactory bulbs and tract, which measured 7.95 cm^2^. The total neocortex surface area was 8.4 cm^2^, hence a neocorticalization of 10%. The total endocast surface area of *M. obtusidens* was 133 cm^2^, with neocortical surface area of 31 cm^2^ and a neocorticalization percentage of 23%. Both endocasts contain a disproportionate amount of hindbrain. The denominator is high in both specimens, reflecting greater representation of hindbrain relative to forebrain.

*Smilodectes*

The endocast of this Eocene lemuroid (shown in **Fig. 10A**) *Smilodectes gracilis* (FMNH PM 56263), was copied from YPM VP 12152. *S. gracilis* is from Uinta County, Wyoming. I was given a copy by C.L. Gazin (1958) but scanned the more carefully prepared endocast in the Radinsky Collection at FMNH, which originates from the Bridger Formation in Wyoming and is dated to 48 Ma. Radinsky and I differed in our views on its body size (Jerison, 1979, Radinsky, 1979). I judged it to be a 1.6 kg prosimian. The endocast scans (length = 3.4 cm) are shown in **Fig. 10A**. The endocast volume *E* = 9 ml; *EQ* = 0.55; endocast surface area (olfactory bulbs missing) = 25.93 cm^2^; neocortex surface area = 9.13 cm^2^; and neocorticalization = 35.23%. The endocast was compared with that of the Paleocene *Plesiadapis tricuspidens* by Orliac et al. (2014), who noted that *EQs* in the later primates were higher, the endocasts more globular, and olfactory tract and bulbs were smaller.

*Notharctus*

This endocast, FMNH PM 59264 and identified only as “Tertiary: Eocene,” is shown in **Fig. 10B**. It is described in Gunnell et al. (2007). Fleagle (1999) estimated the body weight in four species as between 4200 g in *N. tenebrosus* to 6900 g in *N. robustus*, based on tooth measurements. The endocast length (olfactory bulb to medulla) = 5 cm. The lateral view emphasizes the rhinal fissure emerging from the olfactory bulb. The specimen was sketched by Radinsky (1979). My measurements were of the left hemisphere but, despite its distorted form, a reasonable guess of its endocast volume is *E* = 15.38 ml. I used a 4200 g body weight and a 15 ml endocast volume for the analysis of encephalization in the specimen; *EQ* = 0.48. Estimating neocorticalization was difficult because of the fragmented endocast, which was of approximately half the brain region and included posterior “brain” and matrix. An estimate of its total brain surface area is 40.57 cm^2^; half its neocortex area is about 5 cm^2^; neocortex = 10 cm^2^; olfactory bulbs = 2.32 cm^2^; remainder = 38.25 cm^2^; and neocorticalization = 26.14%. Its age is about 47 Ma.

### Later Eocene Fossils

Here, “Later Eocene” is 42 Ma to 32 Ma.

*Necrolemur*

The scanned endocast is of *Necrolemur antiquus*, FMNH PM 59261 copied from YPM VP 18302. This small fossil primate is from the Quercy deposits, late Eocene, about 37 Ma. There are a number of body size estimates, all small. Working from tooth data, Fleagle (1999) calculated body size *P* = 320 g; endocast *E* = 5.05 ml; and *EQ* = 0.90. The endocast is remarkably clean, and somewhat more than half could be cast. The half volume was determined from the clear midline ridge. Its encephalization is the highest of the Eocene mammals. Its olfactory bulbs and tract as distinguished from the rest of the forebrain lead to a reasonable analysis of neocorticalization. The endocast surface area = 39.18 cm^2^; olfactory bulbs = 2.08 cm^2^; remainder = 37.10 cm^2^; neocortex = 14.04 cm^2^; and neocorticalization = 37.84%. The endocasts are shown in **Fig. 10C**; the lateral view of almost all of the left temporal lobe with some rhinal fissure is visible at its lower border. The almost hidden rhinal fissure in lateral views is typical of later primate brains.

*Adapis*

My scan of this Eocene lemuroid (NHMUK M1340 = FMNH 59259), *Adapis parisiensis*, is of Professor R. D. Martin’s (1990) endocast, which has clearer surface features than earlier preparations (LeGros Clark, 1959; FMNH PM 59275). It is from a well-preserved Quercy skull and dates to 34.1 Ma; *E* = 8.2 ml; length (olfactory bulbs to medulla) = 4.7 cm. I estimated its body weight *P* = 1600 g (cf. Jerison, 1979; Radinsky, 1979). The *EQ* = 0.50; endocast surface area = 24.95 cm^2^; olfactory bulbs = 1.42 cm^2^; remainder = 23.53 cm^2^; neocortex = 10.17 cm^2^; and neocorticalization = 43.23%. It is similar in shape to the brains of living lemuroids. My FMNH 59259 is shown in **Fig. 10D**.

My other *Adapis* specimen, FMNH PM 59275, has *E* = 7.85 ml; *P* = 1600 g; *EQ* = 0.48, and is not illustrated. The specimen may occasionally have been referred to as *A. magnus*. The endocast surface area = 26.52 cm^2^; olfactory bulbs = 1.55 cm^2^; remainder = 24.97 cm^2^; neocortex = 13.25 cm^2^; neocorticalization = 53.06%. The data are quite similar for the two specimens; the difference is due to my error of measurement and some minor differences between the endocasts. Martin (1990) provided a detailed review of these specimens and of other fossil and living primate brains and endocasts, including consideration of sexual dimorphism.

*Uintatherium*

I scanned the endocast of *Uintatherium anceps* (YPM VP 11036), prepared by O. C. Marsh, from material retrieved from the Bridger Formation in Uinta County, Wyoming (Marsh, 1886). One of the largest species of its time, it dates from about 49 Ma. My scanned endocasts are shown in **Fig. 11A**. Its body size was determined by scaling up a carefully prepared model (Jerison, 1973) to produce a body weight of about 1,250 kg. The entire endocast measured 434 ml, but when the olfactory bulbs were removed it measured 386 ml. The anterior portion of the skull was almost certainly drilled out when the skull was prepared for the endocast, and the size of the endocast’s “olfactory bulbs” are grossly overestimated. The endocast resembles an enlarged rat brain and reflects the error that enlarged olfactory bulbs are primitive traits, more represented in rats than in humans. As in all the specimens in the neocorticalization analysis, the surface area of these “olfactory bulbs” is subtracted from the area of the entire endocast when determining the neocortex-to-endocast area ratio. Taking *E* = 386 ml as the brain volume, the *EQ* = 0.28. The endocast length as illustrated (including olfactory bulbs) = 17.2 cm; surface area (including olfactory bulbs) = 391.2 cm^2^; olfactory bulbs = 76 cm^2^; remainder = 343.20 cm^2^; neocortex = 64.37 cm^2^; and neocorticalization = 18.75%.

*Megacerops coloradensis*

The endocast of this brontothere (**Fig. 11B**) was probably prepared from a skull from the White River Group. It is catalogued as FMNH PM 59199 and identified only as from the Radinsky Collection, but I think it was copied by Radinsky from YPM VP 12010. In the FMNH catalogue it is listed with a minor spelling error as *Titanotherium “inges” (Menodus)*. In the Yale catalogue it is referred to as Bronthotherium *ingens*, and following Mihlbachler et al. (2004), it is likely synonymous with *Megacerops coloradensis*. It was described by O. C. Marsh (1874) as collected from Colorado, “Devendorf party (1873)” and called early Oligocene. I believe that it would now be dated Late Eocene 34 Ma. Described in various places as small-brained, its impressive endocast, *E* = 750 ml, is of an appropriate size for its time and body size. The *EQ* = 0.25 and length = 17.1 cm. It was a heavily built animal that measured about 4.3 meters in length and weighed about 4,000 kg. The endocast surface area = 521.47 cm^2^; olfactory bulbs = 36.62 cm^2^; remainder = 484.85 cm^2^; neocortex = 164.37 cm^2^; and neocorticalization = 33.9%.

*Moeritherium*

This endocast (**Fig. 11C**) of the early Eocene proboscidian (NHMUK PV M 9176 b) is from the Fayum in Egypt (Fayum Chron17, 37 Ma). The animal has been described as pig-sized or small hippopotamus-sized. Its skull as sketched in Savage & Long (1986) is 44 cm long, and I estimate its body length to be about 200 cm. My formula for a species of heavy habitus of that body length suggests body weight *P* = 394 kg, used here. Savage & Long indicated a lower body weight of 230 kg; I do not know their source. Its endocast *E* = 233.33 ml and *EQ* = 0.36. The reconstructions of the body have an interesting history. One was prepared for the 1851 London Crystal Palace Exposition, and I think it is presently in the entry court of MNHN. The endocast length = 11.6 cm; surface area = 265.93 cm^2^; olfactory bulbs = 34.58 cm^2^; remainder = 231.35 cm^2^; and neocorticalization = 33.8%. **Fig. 11C** shows that the matrix forms a large mass of material at the ventral border of the olfactory bulbs, which can be removed by digital editing.

*Arsinotherium*

*A. zitelli* (NHMUK PV M 8539), suborder Embrithopoda, from the Fayum in Egypt about 33 Ma, was one of the heaviest species of its time. Its endocast is illustrated in **Fig. 11D**. There appears to be matrix added to the right cerebellar area in the region of the flocullus. The most recent description of the species is by Sanders et al. (2010), from which I estimated its heavily built body length as 330 cm. From my Eq. 2, this leads to *P* = 1.51 metric tons. After clipping a portion of its right “cerebellum”, its endocast volume measured 926.6 ml; *EQ* = 0.59; and endocast length = 18.6 cm. Convolutions, presumably present in its brain, did not mold the cranial cavity and are not represented in the endocast. It is unique in my sample of land mammals in that the rhinal fissure is not visible, so neocorticalization could not be estimated.

*Pterodon*

This “creodont” hyaenodontid species, described by Piveteau (1961) as the size of a large wolf, is from the Quercy deposits that date from about 40 Ma to about 32 Ma. My endocasts are shown in **Fig. 12A**. It is one of the “Creodonta”, more specifically a hyainailourine hyaenodontan, that can be compared with the Carnivora illustrated here. This *Pterodon dasyuroides* endocast is NHMUK PV M 25985 b; olfactory bulbs not preserved; length (forebrain to medulla) in the vertically oriented illustration 9.6 cm; and *E* = 58.51 ml. The olfactory bulbs were preserved in the specimen illustrated in Piveteau (1961, p. 809), and the skull illustrated in Piveteau (1961, p. 679) is 23 cm long. I thus estimated the body length to be about 115 cm. This results in a body weight of 37 kg, somewhat less than the 42 kg weight of the wolf recorded by Van Dongen (1998). I date it to 36 Ma. *EQ* = 0.44; endocast surface area = 105.05 cm^2^; neocortex = 37.19 cm^2^; and neocorticalization = 35.4%.

*Stenoplesictis*

Radinsky evidently copied this endocast (**Fig. 12B**) of the Eocene stenoplesictid feliform (FMNH PM 59013) from NHMUK M. 1381, which was a Quercy specimen, probably “*Cynodictis” cayluxi* (now *Stenoplesictis cayluxi*), figured in Piveteau (1961, p. 705 and p. 809). It was a small species of Carnivora, family Stenoplesictidae., with an age of about 34Ma for the scanned specimen. Its skull as pictured in Piveteau was 9.9 cm long, and its body length was about 49 cm, leading to body weight of *P* = 2.8 kg. The endocast volume *E* = 11.60 ml; *EQ* = 0.49; endocast surface area = 33.29 cm^2^; olfactory bulbs = 2.50 cm^2^; remainder = 30.79 cm^2^; neocortex = 12.54 cm^2^; and neocorticalization = 40.73%.

*Cynohyaenodon*

This small “creodont” hyaenodontid is specimen FMNH PM 57153, but there is no record of species or location recorded at FMNH. It is a Quercy locality specimen, about 34 Ma, probably *Cynohyaenodon cayluxi*. Only its left half was preserved and it is shown in **Fig. 12C**, to the left of its lateral view. Although the rhinal fissure was unclear in this image, it was clear enough to estimate neocorticalization. As with all Quercy specimens, Piveteau provided an overall description, and the natural endocast is described by Dechaseaux in Piveteau (1961 p.809, fig.189). Piveteau included information on skull and body size: skull length is given as 2/3 natural size, but from data on the endocast, the actual magnification of the text image was 0.61. I assumed a body length of 61 cm and body weight of *P* = 5.4 kg. My scan of its endocast indicated *E* = 11.04 ml; *EQ* = 0.30; endocast surface area = 32.46 cm^2^; olfactory bulbs = 4.58 cm^2^; remainder = 27.88 cm^2^; neocortex = 8.32 cm^2^; and neocorticalization = 29.83%.

*Quercygale*

My specimen was originally identified as *Procynodictis angustidens*, FMNH PM 57168, and was copied from AMNH FM 95590, probably by Radinsky (1978). Its endocast volume *E* = 23.33 ml. According to the AMNH catalogue, it is *Quercygale angustidens* from the Quercy deposits in France. McKenna & Bell (1997) suggested that *Procynodictis* may be a *nomen nudum*, but Radinsky, who earlier examined the skull at MNHN, chose the listed name for the FMNH specimen. Piveteau (1961) evidently accepted the name. *Procynodictis* is listed among the Canidae in McKenna & Bell (1997), whereas *Quercygale* is listed among the Viveridae, and later assigned to Miacidae (Wesley-Hunt and Werdelin, 2005), although there is still some uncertainty in its precise phylogenetic position within Carnivoraformes (e.g., Tomiya, 2011). The distinction may be appropriate for paleoneurologists who study the details of patterns of convolutions as Radinsky does, but I am skeptical about the emphasis on those convolutional details as firm signs of functional areas. The  *Quercygale* endocast is shown in **Fig. 12D**; in the view of its lateral surface, the rhinal fissure is faintly visible dorsal to the olfactory tract. The endocast length = 6.3 cm. Radinsky (1978 p. 828) reported a body size of about 6 kg, which he “modeled after other carnivores”. I found no other estimates and used *P* = 6571 g. Radinsky suggested a geological age of about 40 Ma, the older extreme for Quercy; he may have used the probable age of the American species of *Procynodictis*. In any case, an age of 40 Ma is an acceptable estimate and it is used here. The *EQ* = 0.55, which may be high for the Eocene and suggests that the body size is underestimated. The endocast surface area = 53.09 cm^2^; olfactory bulbs = 1.32 cm^2^; remainder = 51.77 cm^2^; neocortex = 21.56 cm^2^; and neocorticalization = 41.64%, also a relatively high value for an Eocene species, in this instance uninfluenced by the estimate of body size.

*Cebochoerus*

The skull of *Cebochoerus lacustris* illustrated by Piveteau (1961, p. 900. Fig. 14) was 16.4 cm long, indicating a body length of 70 cm. My weight-length equation resulted in a body weight estimate of *P* = 8 kg. My scanned endocast specimen FMNH PM 59051 has not been identified with respect to species or locality, but from Piveteau, *C. lacustris* is the likely name of the FMNH specimen. It is an early relative of the suids (family Cebochoeridae) from the Quercy deposits. Erfurt & Métais (2007) described the genus as from MP13 to MP19 and a geological age of 38 Ma. Only a partial endocast was available, illustrated in **Fig. 13A** and with length = 5.6 cm. Its half “brain” volume indicated a total *E* = 11.9 ml; *EQ* = 0.25; endocast surface area = 34.1 cm^2^; olfactory bulbs = 3.84 cm^2^; remainder = 30.26 cm^2^; neocortex = 7.01 cm^2^; and neocorticalization = 23.17%.

*Hylomeryx*

This artiodactyl endocast is FMNH PM 59055 and was copied from CM VP 2915, originally designated as a paratype for *Sphenomeryx quadricuspis* (Peterson, 1919). The taxon has since been included in *Hylomeryx* (Stucky, 1998),. The endocast was from Uinta C, Member Myton, and its age ranges from 45.9 to 39.5 Ma. It is shown in **Fig. 13B**, olfactory bulbs missing; and length = 4.3 cm. For neocorticalization analysis, I date it to 42 Ma. I found the estimate *P* = 6 kg in my data sheet but do not know its source, and a literature search did not help. The endocast *E* = 9 ml. Assuming that the brain and body size are correct, *EQ* = 0.23; endocast surface area (olfactory bulbs absent) = 26.78 cm^2^; neocortex = 6.71 cm^2^; and neocorticalization = 25.04%.

*Mixtotherium*

The specimen is FMNH PM 59052, with no species or locality data. In his discussion of its 14 cm skull, Piveteau (1961, p. 901, Fig. 15) treated this small artiodactyl as similar to *Cebochoerus* and probably another Quercy species of about 40 Ma. Piveteau called it *Mixtotherium cuspidatum* and described it as another suid-like species, family Mixtotheriidae. It is shown in **Fig. 13C**. Just over half of the endocast could be scanned, and the olfactory bulbs were preserved; length = 6.7 cm; and *E* = 21 ml. Assuming a body length of 62 cm, it weighed *P* = 6 kg; *EQ* = 0.53; endocast surface area = 45.93 cm^2^; neocortex = 12.74 cm^2^; and neocorticalization = 27.73%.

*Chadronia*

The endocast of this species, AMNH FM 109412 (= FMNH PM 57129), is a good image of the brain, despite the shattered skull from which it was prepared. It is the species described by Cook (1954), *Chadronia margaretae*; the species name memorializes his wife, who found the skull. Endocast *E* = 28.86 ml. The skull length was 15.1 cm, indicating a body length of 68 cm and body weight *P* = 7.5 kg. It is late Eocene, about 35 Ma. The animal, according to McKenna & Bell (1997), was a cimolestid and among the Pantolestinae, and later removed to Pantolestidae outside of Pantolestinae (Gunnell et al., 2008). McKenna, amused, once showed it to me as an unusually large and modern looking fossil insectivore endocast, given the frequent view of living insectivores such as hedgehogs (*Erinaceus europeaus*) as having primitive brains. The *Chadronia* endocast is appropriately convoluted for a brain its size, comparable to capybara, and more convoluted than the surprisingly smooth-brained beaver. It had been considered a “creodont” and a mesonychid, and most recently a pantolestid. Regardless, its habits were probably those of a small carnivorous species. It is shown in **Fig. 13D**: length = 7.7 cm; *EQ* = 0.63; endocast surface area = 66.47 cm^2^; olfactory bulbs = 6.09 cm^2^; remainder = 60.38 cm^2^; neocortex = 23.48 cm^2^; and neocorticalization = 38.89%.

*Anoplotherium*

This artiodactyl endocast was recognized by Edinger (1975) as a “first” in the history of paleoneurology. Well-articulated remains of the animal were found in the plaster of Paris gypsum beds of Montmartre. Georges Cuvier named it *Anoplotherium commune*. Part of the natural endocast was mentioned by Cuvier in his 1804 lecture (Cuvier, 1835), when he used this MNHN specimen to discuss fossils. His point was that these were petrified bones, not ordinary rocks that God had shaped like bones to tempt people to adopt views inconsistent with the book of Genesis. In a public demonstration, Cuvier, as organizer and director of the Muséum Nationale d'Histoire Naturelle (MNHN), exposed parts of the fossil and predicted that additional preparation would reveal features evident in the skeletons of living species. With hammer and chisel he exposed previously hidden parts of the skeleton. Opening the cranium revealed part of a convoluted surface of the brain-like endocast. The specimen that I scanned, *Anoplotherium commune* (NHMUK PV M 3753; my **Fig. 14A**), is from Quercy rather than Paris (Palmer, 1913) and is now dated to 34.1 Ma. In an earlier publication (Jerison, 1973), I estimated its endocast volume as 80 ml and its body size as 82 kg. Editing the endocast to remove matrix artifacts from the base of the “medulla”, I remeasured it as *E* = 78.37 ml; *EQ* = 0.35; and length = 10.4 cm. It was an early artiodactyl, family Anoplotheriidae. The endocast surface area = 129.18 cm^2^; olfactory bulbs = 6.88 cm^2^; remainder = 122.31 cm^2^; neocortex = 34.83 cm^2^; and neocorticalization = 28.48%. This endocast is an important example of the railroad-car appearance, elongated to distinguish cerebellum from forebrain as occurs in some Paleogene fossil endocasts.

*Patriomanis*

This Eocene pangolin, *Patriomanis americana* (AMNH FM 78999 = FMNH PM 57103), is listed in the FMNH catalogue as Oligocene, from Bates Hole, Wyoming. Its skull (Emry, 2004) was about 10 cm long, and from Emry’s sketch of its body (Emry and Gaudin, pers. comm.), I determined its head+body length as about 50 cm. It is from the Ash 5 Layer and dates to about 34.7 Ma. My weight-length regression analysis resulted in a body weight of about 3 kg. The scans are shown in **Fig. 14B**. Its endocast *E* = 11.21 ml and *EQ* = 0.45. Interestingly, one living pangolin, *Manis javanica*, has been reported (Van Dongen, 1998) with a body weight of 3.5 kg and brain weight of 11 grams. Evidently there was no progress in relative brain sizes in pangolin species from the Eocene to the present. The endocast surface area = 33.86 cm^2^; olfactory bulbs = 4.21 cm^2^; remainder = 29.65 cm^2^; neocortex = 5.23 cm^2^; and neocorticalization = 17.65%.

*Poebrotherium*

The endocast (AMNH F:AM 31700 = FMNH PM 59167) shown in **Fig. 14C** is from the Brule Formation, Wyoming. I found no definite dating, but from Woodburne (2004) it appears to be late Eocene, about 35 Ma. Multiple regression analysis (Mendoza et al., 2006) gave body weights ranging from 29.8 kg to 42.9 kg. Savage & Long (1986) reported a skull length of 17.5 cm, suggesting a body length of 88 cm. My length-weight equation suggested a much smaller body weight *P* = 16 kg, but I use Mendoza’s lower body weight of 29.8 kg to assess its encephalization. Endocast *E* = 47.82 ml; *EQ* =0.41; length (olfactory bulbs not preserved) = 7.5 cm; surface area = 81.59 cm^2^; neocortex = 33.79 cm^2^; and neocorticalization = 41.41%.

*Bathygenys*

I reviewed one specimen (TMM TXVP 40209-431, and Fig 46 Species 431) from a larger sample of *B. reevesi* described by Dr. Jack Wilson (Wilson, 1971) and more recently studied by Macrini (2009). Although originally dated to 34 Ma, they are Chadronian, about 37 Ma. My specimen’s skull is about 9.4 cm long. If its body had been shaped like that of its larger later relatives (e.g., *Merycoidodon culbertsoni*), it would have been about 42 cm long, weighing about 2 kg for encephalization analysis. Mendoza et al. (2006) estimated its body size from a multiple regression analysis of tooth dimensions in four specimens, the results showing body sizes ranging between 6 and 8.5 kg, which also seem reasonable. I used 6795 g for my analysis. Its endocast volume *E* = 12.08 ml; length = 5 cm; and *EQ* = 0.28.

The sample of 20 endocasts that Dr. Wilson allowed me to measure provided the best available data at the time (Jerison, 1979) on within-species variability in fossil endocasts. I discuss these data and Macrini’s (2009) analysis in “Within Species Variation” below. In my specimen UT 40209-431 shown in **Fig. 14D**, the olfactory tract abuts the anterior border of the rhinal fissure, but the fissure continues ventral to neocortex. The endocast surface area = 33.46 cm^2^; olfactory bulbs = 2.35 cm^2^; remainder = 31.11 cm^2^; neocortex = 8.71 cm^2^; and neocorticalization = 28.0%.

### Oligocene Fossils

The “Oligocene” samples in this monograph are dated from 32 Ma to 23 Ma. These dates anchor the analysis of neocorticalization as changes with the passage of time.

*Daphoenus*

Two endocasts were identified as *Daphoenus vetus*, YPM VPPU 12588 and FMNH PM UM1; only the former is shown in **Fig. 15A**. They are probably from the White River Badlands in South Dakota and date to 32 Ma. They are from the bear-like arctoid family Amphicyonidae, order Carnivora. Olfactory bulbs were not preserved in either specimen. The endocast volume of YPM VPPU 12588 was *E* = 46.87 ml and FMNH PM UM1 was *E* = 42.61 ml. From tooth sizes, the body weight for the species has been estimated as 7.84 kg (Legendre & Roth, 1988). From the head and body length of about 1 meter, I estimated their body weights as much heavier, about 24 kg; *EQ* = 0.47 for YPM VPPU 12588 and *EQ* = 0.43 for FMNH PM UM1. For PM 59008a, the endocast surface area = 80.89 cm^2^; neocortex = 39.82 cm^2^; and neocorticalization = 49.22%. For 59008b, the surface area = 84.96 cm^2^; neocortex = 39.10 cm^2^; and neocorticalization = 46.02%. My two casts were almost indistinguishable.

*Dinictis*

The scanned half endocast (**Fig. 15B**) of this early nimravid, *Dinictis felina*, order Carnivora, is FMNH PM 58866, copied by Radinsky from the South Dakota School of Mines Museum of Geology (SDSM) specimen 2431. It is Orellan, from the White River Badlands of South Dakota, 32 Ma. The half endocast displaced 30 ml, indicating *E* = 60.1 ml. Its head+body length was 115 cm leading to a body weight of *P* = 37 kg; *EQ* = 0.45; length = 6.8 cm; endocast surface area = 88.4 cm^2^; neocortex = 42.14 cm^2^; olfactory bulbs = 4.9 cm^2^; remainder = 83.23 cm^2^; and neocorticalization = 50.63%.

*Eusmilus*

The endocast of this nimravid sabretooth, *Eusmilus bidentatus* (FMNH PM 58871), is shown in **Fig. 15C**, in which its ectosylvian complex is compared with that visible in the living cat’s endocast and brain. In living cats, this complex represents most of the auditory neocortex (Johnson, 1990), and it is reasonable to assume that this was also true of their relatives of the late Eocene and early Oligocene. My original *Eusmilus* is a Quercy specimen at MNHN and is illustrated in Piveteau (1961, Fig. 202, p. 817). Dating is imprecise; I assumed that it is early Oligocene, partly because individuals from North America are early Oligocene and about 32 Ma. Only about half of the brain is present in the right hemisphere in **Fig. 15C**. The endocast length = 7.2 cm. The half volume was 20 ml, indicating a 40 ml endocast volume. Its 19 cm skull suggested a head+body length of about 90 cm and body weight *P* = 18 kg. Its tooth measurements suggested a much larger animal, perhaps 70 kg (Legendre & Roth, 1982). It has been described as the size of a small leopard (as was *Hoplophoneus*). I propose that *Eusmilus* was about the same size as *Hoplophoneus, P* = 35 kg; *EQ* = 0.31; endocast surface area = 70.19 cm^2^; olfactory bulbs = 4.87 cm^2^; remainder = 65.32 cm^2^; neocortex = 32.92 cm^2^; and neocorticalization = 50.4%.

*Hoplophoneus*

My specimens are USNM Paleobiology V 22538 (**Fig. 15D**) and UM2. In Jerison (1973), I reported two body weights for *Hoplophoneus*: one as *H. oreodontis* and the other as *H. primaevus,* which is shown here. I published a body weight of 20 kg for a 74 cm head+body length in *H. oreodontis*, and a body weight of 49 kg for *H. primaevus* with a 100 cm head+body length. I treated both as of “heavy” habitus. Reviewing skeletal reconstructions, I change these estimates here.

*Hoplophoneus* has been described as the size of a small leopard. Walker (Nowak, 1999) reported the head+body lengths of these living carnivores as 91 to 191 cm, and *H. primaevus* fits into that range. It is also described as heavily built, which may explain my treating its habitus as heavy, although the skeletal reconstructions do not suggest an unusually heavily built small carnivore. Body weights in living leopards are reported as ranging between 37 and 90 kg for males and 28 to 60 kg for females. Van Valkenburgh (1990) estimated body weights of 13 or 19 kg for *H. primeavus* and 66 or 69 kg for *H. occidentalis* using head+body length and skull length in her regression analysis. A reasonable estimate for body size is *P* = 35 kg. The forebrain-to-medulla length = 6.7 cm.

My *Hoplophoneus* specimens are from the South Dakota White River Badlands, early Oligocene, about 32 Ma. Olfactory bulbs have not been recovered. The endocast UM2 volume was *E* = 42.67 ml, hence *EQ* = 0.33. The volume of endocast USNM Paleobiology V 22538 was *E* = 49.47 ml, hence *EQ* = 0.39. Endocast UM2 surface area = 73.11 cm^2^; neocortex = 28.48 cm^2^; and neocorticalization = 39.0%., while endocast USNM 22538 surface area = 79.38 cm^2^; neocortex = 32.57 cm^2^; and neocorticalization = 41%.

The differences between brain measurements in *Eusmilus* and *Hoplophoneus*, including *EQ*, are related to small differences in preservation of the *Hoplophoneus* endocasts and the incomplete endocast of *Eusmilus*, which had to be doubled to provide estimates. They reflect underlying errors in measurements in the material due to the endocasts’ conditions. The errors in these carnivore endocasts were larger than those usually encountered when preparing and analyzing scans.

*Merycoidodon*

The endocast of *Merycoidodon culbertsoni* (FMNH PM UM3) is from the lower Brule Formation of South Dakota. This is no later than 32 Ma, a reasonable date for my specimen. Its head+body length of about 140 cm results in a *P* = 68 kg for its body weight. The scanned endocast did not include an olfactory bulb region. *M. culbertsoni* is one of the most frequently collected Oligocene artiodactyls in the South Dakota Badlands, an oreodont in the family Merycoidodontidae. It is shown in **Fig. 16A**: length (top to bottom) = 7.2 cm; endocast volume *E* = 47.25 ml; *EQ* = 0.24; surface area = 78.05 cm^2^; neocortex = 22.57 cm^2^; and neocorticalization = 28.9%.

*Mesohippus*

This scanned specimen, *Mesohippus bairdi* (AMNH FM 9814 = FMNH PM 59221) from the Brule of the South Dakota Badlands and dated as 32 Ma, was discussed at length and illustrated earlier. The body size determined from Gidley's accurate scale model was *P* = 28.5 kg. The endocast is shown in **Fig. 16B**; length (olfactory bulbs to medulla) = 9.6 cm; *E* = 86.42 ml; *EQ* = 0.77; surface area = 127.48 cm^2^; olfactory bulbs = 9.86 cm^2^; remainder = 117.62 cm^2^; neocortex = 48.89 cm^2^; and neocorticalization = 41.56%. Encephalization in this early Oligocene equid had reached the range of living horses and the evolution of this trait had reached its present grade.

*Promerycochoerus*

My specimen of *Promerycochoerus superbus* (YPM VP 11002 = FMNH PM 59072; **Fig. 16C**) from the John Day Valley, Bridge Creek, dates to about 32 Ma (Woodburne, 2004, p. 330). According to my equation on this heavily built pig-like oreodont, the head+body length = 154 cm and *P* = 178 kg. Smaller body weights were recorded by Damuth & MacFadden (1990), who did not take body build into account. In my specimen, *E* = 147 ml; *EQ* = 0.39; lacking olfactory bulbs, remaining endocast length = 10.8 cm; surface area = 174.4 cm^2^; neocortex = 68.59 cm^2^; and neocorticalization = 39.33%.

*Hesperocyon*

The species is *Hesperocyon gregarius* (FMNH PM 58989) from the Nebraska Brule, dated to about 32 Ma. I estimated its head+body length as 80 cm, indicating *P* = 12 kg. Savage & Long (1986) described it as 45 cm long, which would be only about 2 kg, while Janis (2008) had it as 50 cm. Here I treat it as 50 cm with *P* = 3 kg for encephalization analysis. The species is a member of Carnivora, family Canidae. My endocast in **Fig. 16D** is sectioned at the midline and the data doubled from the measurements; additional errors incurred by having only a partial endocast available and uncertainty about body size must therefore be considered. The measurements suggest a more modern brain than was probably the case; in particular, the cast of the olfactory bulb and tract is unusually large, adding both to endocast volume and to its total surface area. As a result, endocast length = 4.9 cm; volume *E* = 18.8 ml; *EQ* = 0.75; surface area = 37.8 cm^2^; half olfactory bulb area = 2 cm^2^; remainder = 33.75 cm^2^; neocortex = 17.6 cm^2^; and neocorticalization = 52%.

*Leptictis* (*“Ictops”*)

This natural endocast was given to me a half century ago by Dr. James Dye Bump, then Director of the Paleontology Museum of the South Dakota School of Mines near the Badlands of South Dakota. He described it as “*Ictops acutidens* Douglass*”*, and I assume it is from the Badlands Orellan. It had evidently been found as an isolated natural endocast, and it can be dated to about 32 Ma. My scans are shown in **Fig. 17A**. The genus has since been renamed *Leptictis* (Novacek, 1982). My endocast *E* = 3.61 ml and is about half the volume of the *Lepticti*s described by Novacek. I estimated the body size of this small squirrel-sized species as *P* = 500 ml to estimate encephalization, *EQ* = 0.48, which is perhaps high. Endocast measurements for my specimen are length = 3.2 cm; surface area = 15.11 cm^2^; olfactory bulbs = 2.08 cm^2^; remainder = 13.03 cm^2^; neocortex = 1.87 cm^2^; and neocorticalization = 14.3%.

*Leptauchenia*

This oreodont is FMNH PM 59074 (**Fig. 17B**), copied by Radinsky from AMNH FM 627. It is *L. decora* from the Protoceras Beds, Poleside Member of the Brule Formation, White River South Dakota, Whitneyan (31 Ma). The estimated body size is *P* = 39.3 kg (source not traced; possibly from Bill Turnbull, pers. comm.). Measurements of the half endocast were doubled to give an estimated total endocast volume of *E* = 21.95 ml; *EQ* = 0.16; length = 6.3 cm; surface area = 50.78 cm^2^; olfactory bulbs = 5.02 cm^2^; remainder = 45.76 cm^2^; neocortex = 10.35 cm^2^; and neocorticalization = 22.6%.

*Halitherium*

Several copies of the *Halitherium schinzi* plaster endocast were in Tilly Edinger's room at the Senckenberg when I visited Frankfurt in 1999, and one (SMF M 3921) was given to me for my personal collection (**Fig. 17C**). This late Oligocene sea cow had been described by Edinger (1933). *Halitherium schinzi* is from the Egyptian deserts, probably the Fayum, and for the analysis of neocorticalization it is dated to 25 Ma (Gunnell, pers. comm.). The endocast had a small postorbital extension which I removed. Its volume *E* = 266.7 ml; estimated body weight *P* = 250 kg; *EQ* = 0.56; endocast length = 12.2 cm; surface area = 298.77 cm^2^; total neocortical surface area = 94.59 cm^2^; olfactory bulbs not preserved; and neocorticalization = 31.66%.

*Hapalops*

My endocast of *Hapalops* (**Fig. 17D**) is from LACM, but I have no additional museum identification. I further recorded it by reference to Scott (1937) and Piveteau (1961). Another endocast was prepared and discussed by Dozo (1987). *Hapalops* was smaller than *Nothrotherium* but of similar shape. The estimated head+body length is 1 meter, excluding the tail. It was heavily built with a body volume *P* = 49 kg. Scott (1937) had *H. longiceps* and Dozo (1987) had *H. indifferens* as *Hapalops* sp. There is additional discussion in Piveteau (1958) on the species and by Dechasaux (1958) on the endocast. It is Santa Cruzian in age, 17 Ma, from Argentina. Endocast length = 7.8 cm; *E* = 54.7 ml; *EQ* = 0.34; endocast surface area = 91.80 cm^2^; olfactory bulbs = 7.29 cm^2^; remainder = 84.51 cm^2^; neocortex = 30.12 cm^2^; and neocorticalization = 35.6%.

*Leontinia*

Two of my Neotropical notoungulates from the South American Oligocene are shown in **Fig. 18**. One is the toxodon, *Leontinia gaudryi,* shown in **Fig. 18A**. It was a heavily built animal, with a 207 cm head+body length and estimated body weight of *P* = 450 kg. It is dated to 25 Ma, from the Deseado Formation, Pyrotherium beds in Chubut Province, Cabeza Blanca (Loomis loc. J), Rio Chico in Argentina. The endocast is FMNH P 13285; length = 15 cm; endocast *E* = 356.91 ml; *EQ* = 0.51; endocast surface area = 346.90 cm^2^; olfactory bulbs = 28.2 cm^2^; remainder = 318.70 cm^2^; neocortex = 106.48 cm^2^; and neocorticalization = 33.4%.

*Rhynchippus*

The second Neotropical notoungulate is *Rhynchippus equinus* (FMNH P 13410, see Martínez et al. and Dozo & Martínez, 2016), shown in **Fig. 18B**. It was a horse-like notoungulate that was described by Savage & Long (1986) as about 1 meter (head+body) long. Like *Leontinia*, it was from the Deseado of Argentina, about 25 Ma. Body size *P* = 32 kg; endocast volume *E* = 103.56 ml; *EQ* = 0.86; endocast length = 8.9 cm; surface area = 158.13 cm^2^; olfactory bulbs = 15.85 cm^2^; remainder = 142.28 cm^2^; neocortical surface area = 43.14 cm^2^; and neocorticalization = 30.3%.

*Archaeotherium*

The half endocast (FMNH PM 59061) was copied by Radinsky from YPM VPPU 10908 (**Fig. 18C**). It is of *A. mortoni,* an entelodont from the White River beds of South Dakota. I measured the skeleton as 167 cm in head+body length, of heavy habitus, and determined *P* = 230 kg. It was probably from Whitneyan strata, about 32 Ma. Endocast length = 16.2 cm. Endocast doubled half measurements *E* = 169 ml; *EQ* = 0.37; surface area = 204 cm^2^; olfactory bulb surface = 12 cm^2^; neocortex = 49 cm^2^; and neocorticalization = 26%.

*Promartes*

The endocast of *Promartes* (**Fig. 18D**), probably *P. olcotti* (FMNH P 25233), was from Wounded Knee, Decker Canyon, South Dakota, Rosebud Formation, early Arikareean, Oligocene, about 28 Ma. Its volume *E* = 24.1 ml and skull length = 10 cm, suggesting head+body length = 50 cm and *P* = 3 kg, which are within the range of living martens. *EQ* = 0.97, about the average for living mammals. Endocast length = 5.2 cm; surface area = 49.16 cm^2^; olfactory bulbs = 3.83 cm^2^; remainder = 45.33 cm^2^; neocortex = 16.96 cm^2^; and neocorticalization = 37.4%.

*Mesocyon*

This early canid is likely *M. coryphaeus* (AMNH FM 6946 = FMNH PM 58979), although it is only identified as Mesocyon in the AMNH database, from the John Day beds, unspecified stratum, but about 25 Ma (**Fig. 19A**). Radinsky measured its skull as 15 cm, indicating a body length of 75 cm. Tooth measurements by Legendre & Roth (1988) led to body sizes of 7.4 kg and 7.19 kg in two specimens. I used head+body length x and body size *P* = 10 kg for encephalization analysis. Endocast length = 7.2 cm; *E* = 36.6 ml; *EQ* = 0.66; endocast surface area = 71 cm^2^; olfactory bulbs = 4.99 cm^2^; remainder = 66.02 cm^2^; neocortex = 26.37 cm^2^; and neocorticalization = 40%. This specimen (as AMNH FM 6946) was also figured most recently by Lyras (2009; see also citations mentioned therein).

### Mio-Pliocene Fossils

I dated this group from 23 Ma to mid-Pliocene, about 3 Ma. Although the earliest hominins could be included here, they are reviewed with later Plio-Pleistocene and recent primates.

*Mustelictis*

The specimen (**Fig. 19B**) is labeled FMNH PM 58907, but there is uncertainty about it. Radinsky's label was misread as *“Mustelictis pireteauvi”*, clearly intending *Mustelictis piveteaui*, recognizing the great French paleontologist, Jean Piveteau and matching a published name in the literature (Lange 1970). It may correspond to PVPH PVQ69−1, the specimen indicated in Lange’s work (1970). The specimen is also labeled as Quercy. Its stratum in the Quercy would have been relatively late, about 32 Ma. However, it is also labeled “Aquitainien de Saint-Gérand-le-Puy” (Piveteau, 1961). The FMNH label (probably Radinsky's) has it as “Aquitanian”. Present American dating has the “Aquitanian” as “equivalent to the latest Arikareean - early Hemingfordian” (Prothero and Wang, pers. comm.), which is early Miocene, about 22 Ma. I therefore present my data on this early procyonid here rather than with my Oligocene cohort. McKenna and Bell (1997) included it in *Pseudobassaris.* Endocast length = 4.4 cm; *E* = 12.61 ml; *P* = 13 kg; *EQ* = 0.19; endocast surface area = 35.32 cm^2^; olfactory bulbs = 3.25 cm^2^; remainder = 32.07 cm^2^; neocortex = 9.04 cm^2^; and neocorticalization = 28.2%.

*Leptocyon*

The endocast of this Miocene canid (FMNH PM 58961, copied from P AMNH FAM 49063) is described as Late Arikareean, suggesting a date of about 20 Ma. The species name was not recorded. My scans are shown in **Fig. 19C**. It is about 35 cm long and its body weight was reported as *P* = 3.26 kg (Legendre & Roth, 1988). Endocast length = 5.2 cm; *E* = 14 ml; *EQ* = 0.54; surface area = 36.4 cm^2^; olfactory bulbs = 2.6 cm^2^; neocortex = 16 cm^2^; and neocorticalization = 47%.

*Merycoidodon bullatus*

The endocast of this oreodont is of *Eporeodon socialis* (YPM VP 13118 = FMNH PM 59076), now synonymized into *Merycoidodon bullatus* (Christopher Norris at Yale, pers. Comm.; Stevens and Stevens 1996). The fossil was described and its mounted skeleton illustrated in Thorpe (1921). It is from Scott's Bluff, Nebraska, Arikareean, dated 23 Ma. My scans are shown in **Fig. 19D**. The endocast length = 7.6 cm; *E* = 41.79 ml; total surface area = 78.72 cm^2^; olfactory bulbs = 6.92 cm^2^; remainder = 71.8 cm^2^; neocortex = 23.67 cm^2^; and neocorticalization = 32.97%. From Thorpe's mounted skeleton, the head+body length = 93 cm, which results in a *P* = 19.4 kg and *EQ* = 0.48.

*Enaliarctos*

The endocast of this early pinneped (FMNH PM 57161), *Enaliarctos sp.*, is dated to approximately 22 Ma and is shown in **Fig. 20A**. The animal was 144-154 cm long. Taking 150 cm as its length, this gives a *P* = 82 kg, which is consistent with the regression estimate by Berta & Ray (1990). The well preserved endocast of *Enaliarctos* is primarily of forebrain, although the olfactory bulbs are missing, as are some hindbrain (cerebellar) extensions. Although details were not recorded, it may correspond to LACM (CIT) 5302, figured by Mitchell and Tedford (1973). Extant California seal (*Zalophus californianus*) females are approximately in the body size range of *Enaliarctos,* 150-200 cm long and 50-110 kg (Nowak, 1999). No brain-body data were available for this species, but records of brains of living seals in the body size range 39.6-150 kg are from 270-442 g (Van Dongen, 1998). Despite its modern appearance, the fraction of brain of *Enaliarctos* indicated by the endocast was *E* = 118 ml; *EQ* = 0.52; endocast length as pictured 7.2 cm; neocortex = 80 cm^2^; surface area = 132 cm^2^; and neocorticalization = 61%, larger than many living mammals. The denominator in the ratio (endocast surface area) was low because of incomplete preservation, so the surface area is very much underestimated in view of the incompletely represented total brain size.

*Potamotherium*

This mustelid *Potamotherium valetoni* (NHMUK PV M 29357 = FMNH PM 58906) was from the Montaigu-le-Bain deposits in France, dating to about 22 Ma. The specimen, including endocast and entire body, were described by Savage (1957). The endocast was also illustrated by Dechaseaux in Piveteau (1961, T. VI vol. 1, p. 815, Fig. 199) and is an example of errors in using published data. It is described as “x 4/3.” My scan of the endocast (**Fig. 20B**) resulted in a length of 5.7 cm. The published photograph at the same position as my scan had a length of 8.5 cm, with a 4/3rds magnification suggesting a true length of 6.4 cm. If I accepted the published photograph’s measurement, my error would be an overestimation of about 10%, not a critical error for my measurements but pointless to retain when much better measurements are available from the digitized data. The endocast *E* = 37.3 ml, and Savage described its head+body length as 29 inches (73.4 cm), hence *P* = 10 kg; *EQ* = 0.67; endocast length = 5.7 cm; surface area = 64.4 cm^2^; olfactory bulbs = 4.02 cm^2^; remainder = 60.37 cm^2^; neocortex = 35.69 cm^2^; and neocorticalization = 59.1%.

*Plesiogale*

This mustelid, *Plesiogale paragale* (NMB M.A.4641), from the Montaigu-le-Bain in France, is dated to 22 Ma. Based on Radinsky’s notes, it could be a cast of = NHMUK MA 4741, although this has not been confirmed. My scans are shown in **Fig. 20C**. Endocast volume *E* = 17.8 ml; *P* = 2 kg; *EQ* = 0.93; endocast surface area = 44 .2 cm^2^; olfactory bulbs = 3.22 cm^2^; remainder = 40.96 cm^2^; neocortex = 16.3 cm^2^; and neocorticalization = 39.8%.

*Zodiolestes*

This Miocene mustelid, *Zodiolestes daimonelixensis* (FMNH P 12032), is from the lower Harrison formation of Sioux County, Nebraska, at the Niobrara River. My scans are shown in **Fig. 20D**. The geology was discussed in Woodburne (2004 p. 211) as late Arikareean, about 21 Ma. Endocast volume *E* = 31.2 ml; *P* = 5 kg; *EQ* = 0.89; endocast length = 6.1 cm; surface area = 61.59 cm^2^; olfactory bulbs = 3.32 cm^2^; remainder = 58.26 cm^2^; neocortex = 29.03 cm^2^; and neocorticalization = 49.85%.

*Desmathyus* (*Hesperhyus*)

I scanned specimen CM VP 1423 (=FMNH M 59066), a tayassuid endocast from the Upper Harrison Formation of Nebraska, dated to 19.5 Ma. My scans are shown in **Fig. 21**. Endocast length = 8.3 cm; *E* = 71.27 ml; body weight *P* = 11.3 kg (source not found); *EQ* = 1.18; endocast surface area = 122.43 cm^2^; olfactory bulbs = 13.59 cm^2^; remainder = 108.84 cm^2^; neocortex = 33.38 cm^2^; and neocorticalization = 30.7%.

*Oxydactylus*

This Miocene camelid endocast of *Oxydactylus longipes* (FMNH P 12117)*,* shown in **Fig. 21B**, is from Agate Springs, Nebraska, and is dated to 19.5 Ma. Damuth (pers. comm.) suggested a body weight of about 250 kg. Endocast length = 10.3 cm; *E* = 86.65 ml; *EQ* = 0.18; surface area = 131.44 cm^2^; olfactory bulbs = 9.24 cm^2^; remainder = 122.20 cm^2^; neocortex = 51.01 cm^2^; and neocorticalization = 41.7%. Dr. Damuth expressed reservations about the body weight which, given the unusual neck and body proportions of the camelid, might be exaggerated, producing the very low *EQ.*

*Homalodotherium cunninghami*

This heavy South American notoungulate endocast (FMNH PM 59291), shown in **Fig. 21C**, was among those cited by Patterson (1937, see also Martínez & Dozo, 2016) and was from the Santacrucian of Patagonia, 17.5 Ma; *P* = about 400 kg. Endocast length = 14.3 cm; *E* = 227.3 ml; *EQ* = 0.35; surface area = 284.85 cm^2^; olfactory bulbs = 15.96 cm^2^; remainder = 268.89 cm^2^; neocortex = 70.22 cm^2^; and neocorticalization = 26.1%.

*Borhyaena*

The endocast of this carnivorous marsupial, *B. tuberata* (FMNH P 13266), shown in **Fig. 21D**, is from San Juan strata in Argentina and is dated to approximately 17 Ma. Argot (2003) reviewed the species in detail for postcranial data, deriving an estimated body weight of *P* = 23 kg based on published regression analyses of similarly built living mammals. From her illustration (Argot, 2003, text-fig. 22) and her tables of data on femoral length, I was able to make a similar estimate based on its head+body length (100.7 cm). My length-weight equation resulted in *P* = 24.6 kg, which I used for estimating encephalization. Endocast length = 7.4 cm; *E* = 43.05 ml; *EQ* = 0.42; surface area = 82.09 cm^2^; olfactory bulbs = 13.08 cm^2^; remainder = 69.0 cm^2^; neocortex = 17.39 cm^2^; and neocorticalization = 25.2%.

*Protypotherium*

This notoungulate endocast *Protypotherium austral* (FMNH P 13046)*,* shown in **Fig. 22A**, was from the coastal San Juan of Argentina and is dated to 16.5 Ma. Its head+body length (Croft & Anderson, 2008) is 74 cm, leading to a *P* = 9.7 kg; endocast length = 5.7 cm; *E* = 16.69 ml; *EQ* = 0.31; endocast surface area = 43.6 cm^2^; olfactory bulbs = 2.61 cm^2^; remainder = 41.01 cm^2^; neocortex = 13.53 cm^2^; and neocorticalization = 32.98%.

*Proterotherium*

The endocast is of *P. cavum* (AMNH FM 9245 = FMNH PM 59742), shown in **Fig. 22B**. It is from the Rio Robles Santa Cruz region, dating to about 17 Ma (Simpson, 1933). Endocast length = 9.2 cm; *E* = 57.35 ml. The body weight was uncertain, hence encephalization was not determined. Endocast surface area = 106.1 cm^2^; olfactory bulbs = 4.54 cm^2^; remainder = 101.55 cm^2^; neocortex = 29.23 cm^2^; and neocorticalization = 28.8%.

*Nesodon*

My endocast is of *Nesodon imbricatus* (FMNH P 13076), shown in **Fig. 22C**, a toxodon from the Santa Cruz formation in Argentina, about 17 Ma. It was described in Patterson (1937). Cassini et al. (2012) reported its body weight as 550 kg, their largest of the Nesodontinae of the Santacrucian age. **Table 1** gives a size of *P* = 250 kg, which I used to estimate encephalization. Endocast length = 12.3 cm; *E* = 180.06 ml; *EQ* = 0.38; endocast surface area = 253.29 cm^2^; olfactory bulbs = 9.28 cm^2^; remainder = 244.01 cm^2^; neocortex = 72.74 cm^2^; and neocorticalization = 29.81%.

*Merycochoerus*

My scan of *Merycochoerus proprius* is shown in **Fig. 22D**. The endocast of this large pig-like oreodont, *Merycochoerus* (AMNH FM 43016 A = FMNH PM 59081), was from the Marsland Formation. “Marsland” is now treated as Anderson Ranch Formation and Running Water Formation (Woodburne 2004, p. 212R), Hemingfordian Formation, about 17 Ma. Thorpe (1937) presented measurements on all of the species that he identified, listing *M. proprius* as the smallest, with a skull length of 28.6 cm, and *M. magnus* the largest, with a skull length of 33.4 cm. He reported an endocast volume of 77 ml, though it is unclear which species he intended; he merely quoted Moodie (1922). I remeasured my endocast (*M. proprius*) and recorded a volume of *E* = 95.74 ml, presumably correcting Thorpe. The head+body length = 150 cm. The skeleton of *M. proprius* was illustrated by Scott (1962, Fig. 220, p. 359) and, from comparisons with skeletal structures illustrated elsewhere, my original index card showed a body length of 120 cm. Body weight *P* = 122 kg; endocast length = 10.2 cm; *EQ* = 0.32; endocast surface area = 142.44 cm^2^; olfactory bulbs = 9.53 cm^2^; remainder = 132.91 cm^2^; neocortex = 39.48 cm^2^; and neocorticalization = 29.7%.

*Adinotherium*

Another toxodon from the Santa Cruz shown in **Fig. 23A** is a smaller species described by Cassini et al. (2012) as weighing 120 kg. The endocast is FMNH P 12986, *Adinotherium ovinum*; *E* = 111 ml; *EQ* = 0.38. It lived about 15.5 Ma. Endocast length = 10.2 cm; surface area = 155.35 cm^2^; olfactory bulbs = 7.99 cm^2^; remainder = 147.35 cm^2^; neocortex = 49.07 cm^2^; and neocorticalization = 33.3%.

*Merychippus seversus*

This endocast LACM (CIT) 2929 (**Fig. S4**). It is probably *Merychippus seversus*, age 15.5 Ma. Its “olfactory bulbs” are atypically large, reflecting destruction of the bony casing of the olfactory bulbs during fossilization. Endocast volume *E* = 259 ml; *P* = 110 kg; *EQ* = 0.94; endocast length = 14.5 cm; surface area = 296.34 cm^2^; olfactory bulbs = 65.09 cm^2^; remainder = 231.25 cm^2^; neocortex = 106.82 cm^2^; and neocorticalization = 46.2%.


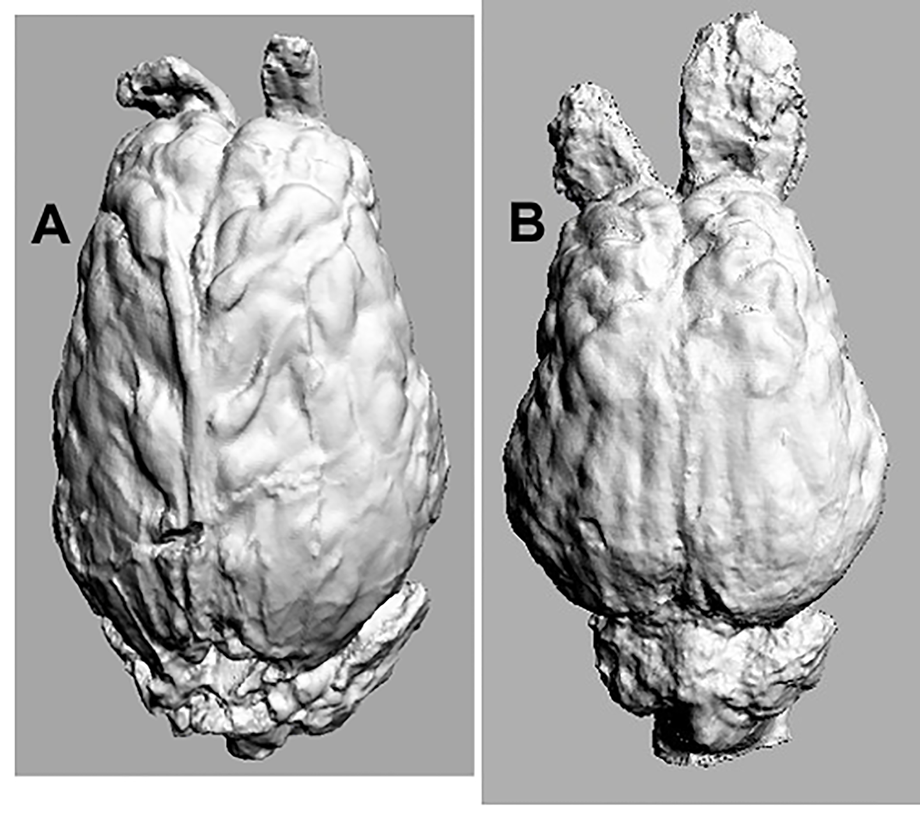


**Figure S4. Olfactory bulbs in *Merychippus.*** Endocasts of two specimens of *Merychippus*. Left-right diameter of each specimen about 25 cm. (A) *Merchippus isonesus* (AMNH FM 71150 = FMNH PM 59208), 232 ml with typical olfactory bulbs. (B) *Merychippus seversus* (LACM (CIT) 2929), 282 ml with enlarged olfactory bulbs, probably resulting from removal of too much bone anteriorly, either naturally or by a preparator.

*Acritohippus isonesus (“Atavahippus”)*

Labeled “*Atavahippus*” in the FMNH catalogue that I used (FMNH PM 59208), Radinsky had evidently copied it from AMNH FM 71150. Unable to locate it in McKenna & Bell (1997), I queried Dr. Susan Bell. She explained, “the name *'Atavahippus'* appears in an unpublished manuscript by Morris Skinner and was applied by him to the endocast in our collection. Because the name was never published, it is not valid -- that is why it does not appear in McKenna & Bell. You should … identify the endocast as belonging to *Merychippus isonesus*”. In 1995, *Merychippus isonesus* was given its own genus, *Acritohippus* (Kelly, 1995). It is from the West Sand Quarry, Lower Snake Creek, Nebraska.

My scans are shown in **Fig. 23B**. Measurements are endocast length = 12.4 cm; *E* = 231.79 ml; *P* = 105.7 kg*; EQ* = 0.86; surface area = 248.43 cm^2^; olfactory bulbs = 18.23 cm^2^; remainder = 230.20 cm^2^; neocortex = 119.03 cm^2^; and neocorticalization = 51.7%.

*Plionictis*

This small early Miocene mustelid is AMNH FM 25314 (= FMNH PM 58945), described in the Radinsky Collection as Barstovian, 15 Ma. My scans are shown in **Fig. 23C**. The body size estimated from tooth data was *P* = 640 g (Legendre & Roth, 1988), an acceptable estimate comparable to living minks and ermines. The specimen was discussed by Loomis (1932). Endocast length = 5.1 cm; *E* = 10.99 ml; *P* = 640 g; *EQ* = 1.23; endocast surface area = 32.97 cm^2^; olfactory bulbs = 2.94 cm^2^; neocortex = 12.63 cm^2^; remainder = 30.03 cm^2^; and neocorticalization = 42.1%.

*Pseudaelurus*

Probably *P. validus*, my scan (**Fig. 23D**) is specimen AMNH FM 61835 (= FMNH PM 58867), one of three endocasts in the Radinsky collection of this genus. No species name was offered. My specimen was described as Early Barstovian, which suggests a geological age of 15 Ma. Radinsky also copied AMNH FAM 61834, recorded as FMNH PM 58870. They are from the Lower Snake Creek Formation of Nebraska. Rothwell (2003) identified it as *P. validus*, and I believe all of the endocasts are of that species. They all feature unusually long olfactory tracts and consequently have enlarged representation of the olfactory system in their brain images. The whole endocast, including olfactory tract and bulbs, was 9.7 cm long and measured *E* = 73 ml. Radinsky had measured the endocast with Archimedes' method as 89 ml. After deleting the olfactory bulbs and tract, the measurement from my digitized scan was *E* = 71.72 ml. I found no record of body size but found *P* = 30 kg in one note, without a source, used here. *EQ* = 0.62; surface area = 114.43 cm^2^; neocortex = 50.38 cm^2^; and neocorticalization = 46.2%.

*Paracynarctus sinclairi*

Recorded in the FMNH catalogue as “*Phlaocyon*”, this canid (AMNH FM 61009 = FMNH PM 58973) is the holotype of *Paracynarctus sinclairi* in Wang, Tedford & Taylor (1999). It was only tentatively recorded by Radinsky as “*Phlaocyon*” when he prepared the endocast (Wang, pers. comm.). The fossil is from Quarry 2, Olcott Formation (Early Barstovian), Sioux County, Nebraska, dated 15.5 Ma, and is shown in **Fig. 24A**. Endocast length = 8.2 cm; *E* = 55.93 ml; *P* = 12263 g; *EQ* = 0.88; surface area = 97.54 cm^2^; olfactory bulbs = 8.53 cm^2^; neocortex = 39.56 cm^2^; remainder = 89.01 cm^2^; and neocorticalization = 44.4%.

*Ustatochoerus*

The endocast (FMNH PM 59071) of this oreodont was prepared by Prof. Len Radinsky from skull AMNH FM 33617, which is recorded as *U. profectus* at the American Museum of Natural History. It was from the Deep Creek Quarry, Brown County, Nebraska, with a geological age of 12.5 Ma. The identification “cannot be shown to differ from *U. major*” (Stevens & Stevens, 2007), and from a sketch of the skull of *U. major*, its head+body length is approximately 100 cm, resulting in a *P* = 24 kg. The endocast, illustrated in **Fig. 24B**, is enlarged in the cerebellar region. I measured its total volume as *E* =162.67 ml, leading to an *EQ* = 1.63, higher than average living species of its body size. In light of the likely significant overestimate of its endocast size, it is reasonable to conclude that it was comparable to living species in encephalization. Endocast length = 10.6 cm; surface area = 209 cm^2^; olfactory bulbs = 11.81 cm^2^; remainder = 197 cm^2^; neocortex = 64.2 cm^2^; and neocorticalization = 32.6%.

*Carpocyon (Osteoborus)*

The endocast of this canid, *Carpocyon webbi* (perhaps still labeled “*Osteoborus tagarctus”* in the Field Museum catalogue) is AMNH FM 61328 = FMNH PM 58964, and was from the Midway Quarry, Burge Member, Valentine Formation, Cherry County, Nebraska (Wang, Tedford & Taylor, 1999). My scans are shown in **Fig. 24C**. Its geological date is about 13 Ma. Its 23 cm skull suggests a head+body length of about 110 cm. My Eq. 2 estimated its body weight as *P* = 32 kg; endocast length = 10.6 cm; *E* = 100 ml; *EQ* = 0.83; endocast surface area = 142.9 cm^2^; olfactory bulbs = 13.1 cm^2^; remainder = 129.8 cm^2^; neocortex = 61.35 cm^2^; and neocorticalization = 47.3%.

*Pseudhipparion*

This specimen is illustrated in **Fig. 24D**. It was listed elsewhere in my notes as *Griphippus* (a former name for the taxon) and is now identified more properly as *Pseudhipparion retrusum* (AMNH FM 70025 = FMNH PM 59211) from Nebraska, Cherry County, Burge Quarry. I date it at 11 Ma. Endocast length = 10.6 cm; *E* = 168.43 ml; *P* = 50 kg; *EQ* = 1.03; surface area = 207.87 cm^2^; olfactory bulbs = 13.39 cm^2^; remainder = 194.47 cm^2^; neocortex = 93.65 cm^2^; and neocorticalization = 48%.

*Paratomarctus*

This Miocene canid, *P. euthos* (AMNH FM 61074) from Burge Quarry, Nebraska (Wang et al., 1999, p. 212), lived about 11 Ma. The genus had been named *Tomarctus* but is renamed in the Wang et al. monograph. I scanned several specimens, and the one illustrated here (**Fig. 25A**) *E* = 56.3 ml; length = 7.6 cm; head+body length = 85 cm; *P* = 10.9 kg; *EQ* = 0.95; surface area = 87.6 cm^2^; olfactory bulbs = 8 cm^2^; remainder = 79.6 cm^2^; neocortex = 35.6 cm^2^; and neocorticalization = 44.7%.

*Hemicyon*

The endocast shown in **Fig. 25B** is of the ursoid *Hemicyon cf.H. barbouri* (AMNH FM 25530 = FMNH PM 59030). It was from Turtle Canyon, lower level, Sheridan County, Nebraska, and was treated as Clarendonian, about 10 Ma. Head+body length = 150 cm; *P* = 82 kg; endocast length =12.6 cm; *E* = 199.28 ml. The allometric expected brain size at that body size was 226 g, hence an *EQ* = 0.88. For comparison, a 143 kg grizzly in my sample had a 234 g brain; its allometric expected brain size was 318 g and *EQ* = 0.74, so *Hemicyon* was a bit brainier than its living relative. Surface area = 251.2 cm^2^; olfactory bulbs = 8.2 cm^2^; remainder = 244 cm^2^; neocortex = 98 cm^2^; and neocorticalization = 40.3%.

Pseudotypotherium

The endocast of this notoungulate from Argentina is AMNH FM 14509 (= FMNH PM 59292), copied by Radinsky (1981). It has been referred previously to *Pseudotypotherium pseudopachygnathum*, and more recently as *Mesotherium pachygnathum* (MacPhee, 2014). The original fossil is from Monte Hermosa and, after consulting with Dr. Maria Teresa Dozo, I date it to 6 Ma. My scans are shown in **Fig. 25C**. Endocast length = 8.6 cm; *E* = 63.7 ml; *P* = 80 kg; *EQ* = 0.29; surface area = 104.6 cm^2^; olfactory bulbs = 5.97 cm^2^; remainder = 98.64 cm^2^; neocortex = 48 cm^2^; and neocorticalization = 48.66%.

*Typotheriopsis*

My endocast of *T. internum* (FMNH P 14420) is from the Arroyo Chasico Formation, Huayquerian South American Land Mammal Age, Buenos Aires Province, Late Miocene (Dozo, pers. comm.), about 8 Ma. Dozo described it as a small digging herbivorous species. The endocast of this notoungulate, shown in **Fig. 25D**, is *E* = 75 ml; *P* = 6.9 kg; *EQ* = 1.74; endocast length = 9.6 cm; surface area = 112.8 cm^2^; olfactory bulbs = 4.4 cm^2^; remainder = 108.4 cm^2^; neocortex = 52.69 cm^2^; and neocorticalization = 48.4%.

The genus *Typotheriopsis* may have been misidentified for this specimen. I think of rodents as living small mammals, and the largest of these, capybara, has a 28 kg body weight and 52 g brain in my sample (Crile & Quiring, 1940); *EQ* = 0.47. Although not a digging rodent, capybara would qualify as a model for the notoungulate, and the much larger notoungulate brain of *Typotheriopsis* would not correspond to a 6.9 kg body. The shape of the endocast is appropriate for related notoungulates such as *Pseudotypotherium*, or my sources on its body size may be wrong. Nevertheless, *EQ* = 1.74 may be ignored until this is clarified.

*Cormohipparion*

My endocast of *C. occidental*e (AMNH FM 71886 = FMNH PM 59220) was reported by Radinsky as from the Hans Johnson Quarry in Nebraska, medial Clarendonan in the Miocene, about 10 Ma. The endocast of this Miocene equid was *E* = 364 ml; *P* = 151 kg; *EQ* = 1.07. It is shown in **Fig. 26A**. Endocast length = 11.2 cm; surface area (minus olfactory bulbs) = 309 cm^2^; neocortex = 184 cm^2^; and neocorticalization = 59.5%.

*Procamelus*

The specimen is from the Burge Quarry, estimated age 11 Ma, and is shown in **Fig. 26B**. The volume of the endocast of *P. grandis* (AMNH FM 40425 = FMNH PM 59160) is *E* = 374 ml; *P* = 200 kg; *EQ* = 0.91; endocast length = 14.3 cm; surface area = 366 cm^2^; olfactory bulbs = 16 cm^2^; remainder = 350 cm^2^; neocortex = 131 cm^2^; and neocorticalization = 37.4%.

*Homotherium*

The endocast of the sabretooth *Homotherium* sp. (AMNH FM 95297 = FMNH PM 58891) measured 192.5 ml; I took its body size as *P* = 200 kg; *EQ* = 0.47. My scans are shown in **Fig. 26C**. It was early Pleistocene, about 1.5 Ma. Endocast length = 11.2 cm; surface area (minus olfactory bulbs) = 224 cm^2^; neocortex 128 cm^2^; neocorticalization = 57%.

*Mylodon*

I was given this *Mylodon* endocast during a visit to the Natural History Museum in London (NHMUK) decades ago, but the museum presently has no record either of the endocast or the gift. After many searches, Dr. Pip Brewer, Curator of Fossil Mammals at the museum, suggested checking Woodward (1900) for clues. Indeed, Woodward accurately drew the specimen when he described how and where it was found. My scan is shown in **Fig. 26D**, and the measurements are *E* = 514.88 ml; body weight estimated from a scale model of a similar specimen at LACM gave a *P* = 1100 kg (Jerison, 1973 p. 334); *EQ* = 0.40; endocast length = 15.8 cm; surface area (less olfactory bulbs) = 399.1 cm^2^; neocortex = 119.19 cm^2^; and neocorticalization = 29.86%.

Woodward (1900, p. 64) wrote, “Dr. Rudolph Hauthal, geologist at the La Plata Museum … not only found another piece of skin, but also various broken bones of more than one individual … in a remarkably fresh state of preservation.” The skull must have been lent to NHMUK, where it was “vertically bisected” (Woodward, 1900, p.69) to prepare the endocast. From the description of the cave where it was found, Woodward considered it a subfossil, no more than 0.01 Ma. The original endocast described by Woodward may have been sent to the La Plata Museum in Argentina. I have given my copy of the endocast to LACM, where it is now specimen LACM 157696. The species had been named by Owen (1841).

### Plio-Pleistocene and Recent Fossils

*Glossotherium*

The volume of this endocast (LACMHC 1717-33) of *Paramylodon harlani* from La Brea Tar Pits in Los Angeles was *E* = 502 ml; from a scale model I estimated *P* = 1100 kg (Jerison, 1973); *EQ* = 0.39. It is shown in **Fig. 27A**. Endocast length = 15.5 cm; surface area (less olfactory bulbs) = 423 cm^2^; neocortex = 129 cm^2^; neocorticalization = 30.61%. Its age is about 0.03 Ma. Like all La Brea material, it was reviewed and illustrated by Harris (1992).

*Arctodus (Tremarctotherium)*

The endocast of this short faced bear *Arctodus simus* (*Tremarctotherium*) (FMNH PM 59022, attributed in HJJ’s notes to LACM) (Harris, 1992) was from La Brea Tar Pits in Los Angeles. It is shown in **Fig. 27B**. It dates to about 0.03 Ma. Endocast *E* = 654 ml; body size *P* = 526 kg (Christiansen, 1999); *EQ* = 0.84; endocast length = 16.8 cm; surface area (less olfactory bulbs) = 498 cm^2^; neocortex = 296 cm^2^; and neocorticalization = 59.45%.

*Aenocyon dirus*

The endocast of this dire wolf (LACMHC 2300-82) was from La Brea Tar Pits in Los Angeles. It is shown in **Fig. 27C**, and it dates to about 0.03 Ma; *E* = 181 ml; *P* = 80 kg; *EQ* = 0.81; endocast length = 11.7 cm; surface area (less olfactory bulbs) = 193 cm^2^; neocortex = 114 cm^2^; and neocorticalization = 59.37%.

*Megalonyx*

This specimen of *M. jeffersoni* (Harry Jerison personal collection) is purportedly from the La Brea Tar pits, but the Natural History Museum of Los Angeles County Rancho La Brea collections manager was unable to find records of this specimen. The species is historically interesting because a specimen of this ground sloth was among those named by Thomas Jefferson. It dates to about 0.03 Ma and is shown in **Fig. 27D**. My endocast *E* = 332.78 ml; *P* = 370 kg; *EQ* = 0.54; endocast length = 12.6 cm; surface area (less olfactory bulbs) = 275.75 cm^2^; neocortex = 103.69 cm^2^; and neocorticalization = 37.6%.

*Nothrotheriops*

LACMHC 1800-6 is the endocast of a La Brea ground sloth *N. shastensis* dating to about 0.03 Ma. It is shown in **Fig. 28A**. Endocast volume *E* = 277 ml; *P* = 320 kg; *EQ* = 0.49; endocast length = 11.8 cm; surface area (less olfactory bulbs) = 239 cm^2^; neocortex = 99 cm^2^; and neocorticalization = 41.4%.

*Panthera atrox*

The endocast of *P. atrox (LACMHC 2900-1)*, the La Brea lion, is shown in **Fig. 28B** and dates to about 0.03 Ma. Endocast volume *E* = 338 ml; *P* = 325 kg; *EQ* = 0.60; endocast length = 13.8 cm; surface area (less olfactory bulbs) = 305 cm^2^; neocortex = 167 cm^2^; and neocorticalization = 54.64%.

*Smilodon fatalis*

The endocast is of *S. fatalis* (LACMHC 2001-199), the sabretooth “cat” that is the official fossil of California. It lived about 0.03 Ma and is shown in **Fig. 28C**. Endocast volume *E* = 216 ml; *P* = 250 kg; *EQ* = 0.45; endocast length = 11.7 cm; surface area (less olfactory bulbs) = 240 cm^2^; neocortex = 120 cm^2^; and neocorticalization = 50.11%.

*Urocyon*

The Pleistocene grey fox, *Urocyon cinereoargenteus*, from La Brea Tar Pits (UCMP V 12263), lived about 0.03 Ma. It is survived by living individuals in Southern California and the Channel Islands. Its endocast is shown in **Fig. 28D**. Endocast length = 6.5 cm; *E* = 39 ml; *P* = 5 kg; *EQ* = 1.11; surface area (less olfactory bulbs) = 64 cm^2^; neocortex = 37 cm^2^; and neocorticalization = 58.02%.

*Platygonus*

The endocast of this tayassuid *Platygonus compressus* (CM VP 12888 = FMNH PM 59058) from La Brea dates to 0.03 Ma. Only half of the endocast was preserved, but it was a relatively clean half, easy to double to estimate its measurements. It is shown in **Fig. 29A**. *E* = 130 ml; *P* = 130 kg; *EQ* = 0.42; endocast length = 9.7 cm; surface area (less olfactory bulbs) = 129 cm^2^; neocortex 74 cm^2^; and neocorticalization = 57.71%.

*Sthenurus*

Professor Radinsky collected this endocast in Wellington Cave, New South Wales, and his widow, Dr. Sharon Emerson, donated a cast of the endocast to the Radinsky Collection. The original endocast was supposedly donated to and accessioned at the Australian Museum under catalog number AM F.30652. However, the curator at the Australian Museum cannot find the endocast, and the specimen in that collection that bears the number AM F.30652 is a long bone, not an endocast. It is *Sthenurus cf. orientalis*, specimen FMNH PM 59245, shown in **Fig. 29B**. My software measured its volume as *E* = 107 ml, and from Wells & Tedford (1995) I estimated the geological age as 0.5 Ma. The body size of this extinct large kangaroo was *P* = 200 kg; *EQ* = 0.26; endocast length = 8.4 cm; surface area (less olfactory bulbs) = 130 cm^2^; neocortex = 67 cm^2^; and neocorticalization = 51.82%.

*Thylacoleo*

The endocast of *T. carniflex* (FMNH PM 59244) was copied by Prof. Radinsky from the South Australian Museum P18681 (SAMA P18681); age estimated as 2 Ma. It is shown in **Fig. 29C**. Although I avoid discussing gyral features, in this case it is worth noting that the characteristic gyri of the felid and canid endocasts are not a feature of this marsupial carnivore. In felids and canids, the ectosylvian gyri are useful maps of the auditory cortex; they are not a feature in the marsupial. Endocast volume *E* = 120 ml; body weight of this marsupial sabretooth *P* = 130 kg (Wroe, 2004); *EQ* = 0.39; endocast length = 9.6 cm; surface area (less olfactory bulbs) = 154 cm^2^; neocortex = 64 cm^2^; and neocorticalization = 41.37%.

*Archaeolemur*

The endocast of *Archaeolemur majori* (AMNH FM 30007 = FMNH PM 59258), copied by Radinsky from the AMNH specimen, is shown in **Fig. 29D**. It is a subfossil lemur from Madagascar and can be dated to 0.01 Ma. Endocast volume *E* = 96 ml; *P* = 17 kg; *EQ* = 1.21; endocast length = 7.6 cm; surface area = 122 cm^2^; olfactory bulbs = 1.88 cm^2^; remainder = 119.3 cm^2^; neocortex = 76 cm^2^; and neocorticalization = 63.7%.

*Pachylemur*

This Madagascar lemur (FMNH PM 59253), probably copied from MNHN, is shown in **Fig. 30A**. It was listed as *Lemur insignis* by Radinsky and has been renamed *Pachylemur insignis*. It became extinct only a few thousand years ago, and the endocast may be dated to 0.01 Ma. Endocast volume *E* = 57.4 ml; *P* = 10 kg (Fleagle, 1999, table 4.2); *EQ* = 1.03; endocast length = 6.4 cm; surface area (less olfactory bulbs) = 80 cm^2^; neocortex = 49.61 cm^2^; and neocorticalization = 61.73%.

*Palaeopropithecus*

This endocast is of *P. maximus* (FMNH PM 59250). It may be derived from a skull in the NHMUK collection (NHMUK M 9898), but curatorial staff there were unable to confirm this. My scans are shown in **Fig. 30B**. I estimated the age of this Madagascar subfossil lemur as 0.01 Ma. Endocast volume *E* = 108 ml; *P* = 50 kg; *EQ* = 0.67; surface area (less olfactory bulbs) = 134 cm^2^; neocortex = 73 cm^2^; and neocorticalization = 54.48%.

Australopithecines

My volume measurements on my australopiths are from the literature, not from my scans. Surface areas on my scans were measured from the fairly complete half-brain scans. Lateral images of both australopiths show the essentially complete right half of each endocast.

*Paranthropus robustus*

The scans of *P. robustus* (SK1585) are shown in **Fig. 30C**. I estimated the geological age as 2.5 Ma. Midline positioned vertically, length = 11.6 cm; *E* = 530 ml (Holloway et al., 2004); *P* = 40 kg; *EQ* = 3.78, about 4 times that of average living mammals. Neocortex = 275 cm^2^; endocast surface area (less olfactory bulbs) = 356 cm^2^; and neocorticalization = 77.18%.

*Australopithecus africanus*

This is the famous endocast of *A. africanus*, the specimen from Taung, discovered and described by Raymond Dart (1925). It brought human origins more than a million years earlier than “*Pithecanthropus erectus*” (*Homo erectus*), which had been discussed and described by Eugen Dubois (1895). Dart was criticized by other anatomists and paleontologists when he discussed the specimen, but his discovery was accepted within decades (Tobias, 1971; Falk, 2009). My endocast, **Fig. 30D**, was unlabeled in a drawer at FMNH, but I believe it was copied from Professor Phillip Tobias's collection which is described in several places, most usefully in Holloway et al. (2004). It is described as from a 3-year-old child and assigned specimen number Taung 1.

The reconstructed endocast (including the missing fraction of left hemisphere) estimated an adult *E* = 440 ml; adult body size *P* = 40 kg; *EQ* = 3.13, about the same as living chimpanzees. Endocast length = 10.8 cm. The endocast fragment illustrated in **Fig. 30D** was 278 ml in volume, but for encephalization I took the accepted adult *E* = 440 ml. Surface area = 242 cm^2^; neocortex = 195 cm^2^; and neocorticalization = 80.54%. Although this specimen is subadult, the neocorticalization probably accurately reflects the adult state. It is dated to about 3.5 Ma.

### Cetacean Fossils

As in the living cetacean brain, there is no rhinal fissure in the fossils and thus no indication of an olfactory bulb or tract; therefore, neocorticalization cannot be assessed.

Scans of my three fossil whales are shown in the left panel of **Fig. 31**; forebrain at the top in each case. These are discussed at length in Jerison (1973, pp. 346-351), and two more recent publications (Gingerich, 2016; Uhen, 2004) extend that discussion. The right panel of **Fig. 31** shows photographs of a dolphin brain (University of Wisconsin specimen 66-130, *Tursius truncatus*) to compare with the fossils. The top and center dolphin brain photographs have the forebrain pointing left. The bottom dolphin brain is a ventral view with forebrain to the right. The optic chiasma is evident with unclear olfactory bulbs and tract.

*Dorudon*

*Dorudon atrox,* named *Basilosaurus* and *Zeuglodon* in earlier reports, is NHMUK PV M 10173 b. It was from the Fayum in Egypt, about 40 Ma. My scan is shown in the top of the left panel of **Fig. 31**. In Jerison (1973), I guessed its weight as 20,000 kg, probably based on its long skeleton as mounted in Egypt, or perhaps from the discussion by Breathnach (1955). Uhen (pers. comm.) indicated a more likely weight of *P* = 394 kg based on a model he had prepared from the Egyptian skeleton. *E* = 460 ml; length (forebrain to medulla) = 13.1 cm. The endocast has a good deal of non-neural material but, taking it all as “brain” and *P* = 394 kg, *EQ* = 0.71.

*Argyrocetus*

The endocast of *A. joaquinensis,* a Miocene dolphin (USNM 11996), dates to about 17 Ma. My scan is shown as the center of the left panel in **Fig. 31**. The endocast was found by Dr. Larry Barnes of LACM, but no species name was available when he allowed me to photograph the endocast (Jerison, 1973, fig. 15.4). I wrote that *Argyrocetus* was much smaller than the living bottlenose dolphin (Jerison, 1973), although I had no good estimate of its body size. Uhen (pers. comm.) identified it as USNM 11996 and suggested a body weight of *P* = 80.1 kg, which I now use. I now use *EQ* = 3.01 for the fossil, comparable to living dolphins; length (forebrain to hindbrain) = 13.3 cm; *E* = 630 ml; neocorticalization not measurable.

*Aulophyseter*

I scanned my endocast of *Aulophyseter morricei* (USNM 11230) and it is shown in the bottom of the left panel of **Fig. 31**; age 17 Ma. It was a small sperm whale. Endocast length (neural protuberance to hindbrain) = 16.7 cm; *E* = 2246 ml. Uhen identified it as USNM 11230 (which I now use to identify the specimen) and gave its body weight *P* = 8509 kg; *EQ* = 0.45.

Encephalization in whales is not really comparable to that in land mammals. Whales adapted to life in the water and had few gravitational constraints on their body size. Therefore, size relationships between brain and body do not equate to the relationships in land mammals, in which body size estimates the size of the map of the perceptual world created by the brain.

### Living Non-Primate Mammals

*Aonyx*

My endocast of the clawless otter, *A. (Amblyonyx) cineria*, is Specimen 358 given to me by Professor Len Radinsky. My scans are shown in **Fig. 32A**. The volume of the endocast *E* = 41 ml, and from Nowak (1999) I estimated its body size *P* = 3 kg; *EQ* = 1.63. Endocast length = 6 cm; surface area (less olfactory bulbs) = 67.57 cm^2^; neocortex = 44.62 cm^2^; and neocorticalization = 66.04%.

*Ursus*

I assumed that this black bear endocast from LACM was *U. americanus*, but I have no additional identification. My scans are shown in **Fig. 32B**. Endocast *E* = 277 ml; assume *P* = 140 kg; *EQ* = 0.86; length = 11.8 cm; surface area (less olfactory bulbs) = 262 cm^2^; neocortex = 160 cm^2^; and neocorticalization = 61%.

*Canis latrans*

My scan of a fossil coyote endocast from La Brea Tar Pits (LACMHC 3200-7) is shown in **Fig. 32C** but is not represented in Table 1. The age of this specimen is about 0.03 Ma. Endocast *E* = 98 ml; the body size was assumed to be the same as its living relatives, *P* = 15 kg; *EQ* = 1.34; length = 8.4 cm; surface area (less olfactory bulbs) = 110 cm^2^; neocortex 74 cm^2^; and neocorticalization = 67.3%.

My coyote braincastwas from the Wisconsin brain collection, which is now housed in the National Museum of Health and Medicine (NMHM Vertebrates WISC 62-301). Its data are in **Table 1**. Brain volume *E* = 72.67 ml; *P* = 15 kg; *EQ* = 1.0; surface area 125.27 cm^2^; neocortex = 82.44 cm^2^; olfactory bulbs = 8.22 cm^2^; remainder = 117.05 cm^2^; and neocorticalization = 70.43%.

*Felis catus*

This endocast of *F. catus* was a gift from Professor Radinsky and is his specimen 101, casted from FMNH Mammals 146456. My scans are shown in **Fig. 32D**. Endocast volume *E* = 25.41 ml; assuming a small domestic cat weight, *P* = 3 kg; *EQ* = 1.02, average for living mammals; endocast length = 5.5 cm; surface area (less olfactory bulbs) = 49.31 cm^2^; neocortex = 28.87 cm^2^; and neocorticalization = 58.54%.

*Cerdocyon thous*

This is Prof Radinsky’s Specimen 294 (AMNH Mammals 36501 = FMNH Mammals 146294 = LBR/Rad 294). My scans of the endocast of this crab-eating fox *C. thous* are shown in **Fig. 33A**. Endocast volume *E* = 45.67 ml; assuming a body weight *P* = 6 kg, *EQ* = 1.15; length = 7.1 cm; surface area (less olfactory bulbs) = 71.57 cm^2^; neocortex = 43.89 cm^2^; and neocorticalization = 61.32%.

*Odocoileus*

I scanned the braincast (not endocast) of the white-tailed deer, *O. virginianus* (University of Wisconsin Brain Collection 67-81), which provides significantly more detail. My scans are shown in **Fig. 33B**. Approximately half of the braincast was available, so doubling resulted in *E* = 124.6 ml. Assuming *P* = 75 kg; *EQ* = 0.58; brain length = 11.2 cm; surface area = 206.58 cm^2^; olfactory bulbs = 6.6 cm^2^; remainder = 199.98 cm^2^; neocortex = 102.24 cm^2^; and neocorticalization = 51.13%.

My record on this deer is uncertain; I made different measurements over several years which resulted in some small discrepancies between two sets of measurements on one specimen.

*Ursus (Kodiak)*

My endocasts of this Kodiak bear from LACM are shown in **Fig. 33C**. Endocast volume *E* = 488.55 ml; estimated body weight from Nowak (1999) *P* = 700 kg; *EQ* = 0.52; length = 18.5 cm; surface area (less olfactory bulbs) = 441.64 cm^2^; neocortex = 224.44 cm^2^; and neocorticalization = 50.82%.

*Lama*

I scanned the braincast (not endocast) of Specimen 65-139 (*Lama glama)* of the University of Wisconsin brain collection, which is now housed at the National Museum of Health and Medicine (NMHM Vertebrates WISC 65-139). My scans are shown in **Fig. 33D**. Endocast volume *E* = 172.22 ml; *P* = 150 kg. Again, note how much more detail shows on a brain than on an endocast. Brain length = 11 cm; surface area remainder = 226.74 cm^2^; neocortex = 144.2 cm^2^; and neocorticalization = 63.6%.

*Lutra lutra*

My possibly erroneous record has this as the European species *L. lutra*. My scans of Radinsky's Specimen 366 are shown in **Fig. 34A**. Endocast volume *E* = 39.22 ml; *P* = 10 kg; *EQ* = 0.70; endocast length = 6.6 cm; surface area (less olfactory bulbs) = 68.58 cm^2^; neocortex = 40.6 cm^2^; and neocorticalization = 59.2%.

*Lontra canadensis*

My scans of Radinsky's otter Specimen 129, probably *L. canadensis (FMNH Mammals 146394),* are shown in **Fig. 34B**. Endocast volume *E* = 59.87 ml; *P* = 10 kg; *EQ* = 1.07; endocast surface area (less olfactory bulbs) = 89.63 cm^2^; neocortex = 54.43 cm^2^; neocorticalization = 60.73%. This was probably the American species. Its endocast is impressively larger than my other *Lutra* species.

*Procyon lotor*

Radinsky's endocast specimen 154 (FMNH Mammals 146352 = AMNH Mammals 8335) is of the raccoon *P. lotor.* My scans are shown in **Fig. 34C**. Endocast volume *E* = 54.18 ml; *P* = 7 kg; *EQ* = 1.23; endocast length = 7 cm; surface area (less olfactory bulbs) = 79.23 cm^2^; neocortex = 47.03 cm^2^; and neocorticalization = 59.36%.

I scanned the left hemisphere of a raccoon brain from the Wisconsin Brain Collection (61-824) and show it in **Fig. 34D**. The raccoon brain measurements, no olfactory bulbs, are *E* = 25.79 ml (obviously low and probably shrunken); *P* = 7 kg; brain scan surface area = 60.99 cm^2^; neocortex = 34.37 cm^2^; and neocorticalization = 56.35%.

*Nasua narica*

Wally Welker, late Professor of Physiology at the University of Wisconsin, maintained the important brain collection (<http://www.brainmuseum.org/>) in his department with the cooperation of his associate, Professor John Johnson of Michigan State University. It is a facility I used frequently when comparing my endocasts with brains. Wally gave me casts of brains of over 200 species of mammals, including the raccoon (WISC 61-824) and the coati *Nasua narica* (WISC 58-360 and WISC 62-404). In **Fig. 34**, the scans of the raccoon (**Fig. 34**) and coati (**Fig. 34E**) brains are compared. These are not endocranial casts; they are of brains and show their external appearance vividly, although sulcal depths are obscured when casting whole brains. It is clear that although these brains are similar, they are not identical, and it is difficult to make localizations. Electrophysiological analysis provided a brain map of the coati rhinarium, showing that its nose sensing area was relatively enlarged, and a map of the forepaw area of the raccoon brain is also relatively enlarged. In these experiments, the nose and forepaw areas were stimulated, and electrical responses were recorded at the exposed brain. The electrophysiological maps (Welker, 1990, Fig. 53) show where evoked potentials were recorded on the brains. As mapped physiologically, the coati’s nose area and the raccoon’s forepaw area are dramatically different, each enlarged appropriately, and reflect the animal’s food foraging behavior.

*Phascolarctos*

In **Fig. 35A**, different orientations of the koala endocast are shown. Both braincast and endocast were given to me by Dr. Maciej Henneberg, Professor of Anatomy, University of Adelaide, Australia, and they are deposited in the brain collection at the University of Adelaide. The endocast volume *E* = 36.5 ml; brain volume is 15.93 ml. Encephalization with respect to the endocast, *EQ* = 0.66; with respect to brain, *EQ* = 0.29. Endocast length = 7.2 cm; surface area (less olfactory bulbs) = 71.49 cm^2^; neocortex = 21.32 cm^2^; and neocorticalization = 29.83%. The braincast measurements are hardly comparable to those on the endocast because so much midbrain surface is measurable. Nevertheless, the brain measurements were surface area = 71.57 cm^2^; olfactory bulbs = 2.46 cm^2^; remainder = 69.11 cm^2^; neocortex = 19.57 cm^2^; and neocorticalization (brain) = 28.32%.

*Macropus*

Professor Jack Johnson of Michigan State University prepared my braincast, not endocast, of this kangaroo, as shown in **Fig. 35B**. He informed me that the animal was probably a juvenile. It is *Macropus fuliginosus*, Michigan State University Specimen MSU 64023. I doubled half measurements to estimate *E* = 34 ml. MSU has a recorded brain weight *E* = 61.35 g, which I did not use. Its weight may have been measured at dissection, whereas mine is based on a braincast from a shrunken brain. Brain length = 7 cm. Body weight *P* = 23.6 kg; *EQ* = 0.34. Brain surface area (less olfactory bulbs) = 86.05 cm^2^; neocortex = 38.38 cm^2^; and neocorticalization = 44.6%. The specimen should be compared with the large fossil kangaroo *Sthenurus* in **Fig. 29B**.

*Vombatus*

My data on the wombat (*Vombatus ursinus*) are from a skull donated by E. Allen and sent by Erica Moe (NMV C7780). It was a male. My measurements were based on the half endocast shown in **Fig. 35C**, doubled as appropriate. Endocast length = 7 cm; *E* = 82.2 ml; *P* = 28 kg, based on Nowak (1999), which leads to *EQ* = 0.74. Endocast surface area (less olfactory bulbs) = 103.23 cm^2^; neocortex = 48.45 cm^2^; and neocorticalization = 46.93%.

*Taxidea*

The endocast of this badger, *T. taxus*, Professor Radinsky's Specimen 360, measures *E* = 60 ml, and from Nowak (1999) I assumed a body weight of *P* = 10 kg; *EQ* = 1.08. My scans are shown in **Fig. 35D**. Endocast length = 7 cm; surface area (less olfactory bulbs) = 80.28 cm^2^; neocortex 48.3 cm^2^; and neocorticalization = 60.16%.

### Living Primates

Most of my specimens in this section are from Professor Dean Falk’s collection of primate endocasts (Falk, 1992, 2012 and pers. comm.). She did not report body sizes, and for this section I take most from Van Dongen (1998) and Nowak (1999). At Falk’s request, I gave her endocasts to Dr. John Fleagle at SUNY. His identifications and numbering are included in my description of each endocast. Dr. Sharon Bell at AMNH informed me that they had been deposited in the living mammal collection at AMNH (Bell, Fleagle, pers. comm.), and Fleagle’s numbers refer to that collection. In the set as illustrated in **Fig. 36 and 37**, the brains were shrunken but the endocasts were not.

The endocast of the small South American grey saki *Chiropotes albinasa* is shown in **Fig. 36A**, and the endocast of the much larger African *Mandrillus sphinx* is shown in **Fig. 36B**. The ventrolateral view exposes more of the rhinal fissure, though it is not easy to trace it in this figure; the fissure is often hidden in more familiar lateral views in primates. The saki and mandrill endocasts are similar in shape but differ in the amount of gyrification, presumably related to their size difference; sakis weigh about 2 kg while male mandrills may weigh 20 kg or more. As similar as they are, they are from two distinct groups of primates, the New World Superfamily Callitrichoidea (=Platyrrhini) and the Old World Superfamily Cercopithecoidea (= Catarrhinae).

*Chiropotes albinasa*

The volume of the endocast (FM 94927) of this South American white-nosed bearded saki shown in **Fig. 36A** is *E* = 53 ml. I have no data on its body size, but from Nowak (1999) I took its weight as *P* = 3 kg; *EQ* = 2.12; endocast length = 5.9 cm; surface area = 82.57 cm^2^; neocortex = 65.26 cm^2^; and neocorticalization = 79.03%.

*Mandrillus sphinx*

The endocast of *M. sphinx* AMNH Mammals 274 is shown in **Fig. 36B**. Body size *P* = 18 kg; endocast length = 8.1 cm; *E* = 131.85 ml; *EQ* = 1.60; surface area = 154.88 cm^2^; neocortex = 119.09 cm^2^; and neocorticalization = 76.89%.

*Homo sapiens*

**Fig. 36C** shows the first of my two *H. sapiens* endocasts (Falk A) in this group from Dean Falk’s collection. I have no museum number. This is an unusually small endocast, *E* = 945.7 ml. Assuming small body size, *P* = 50 kg; *EQ* = 5.81; endocast length = 14.3 cm; surface area = 540.59 cm^2^; neocortex = 432.55 cm^2^; and neocorticalization = 80.01%.

*Homo sapiens*

This endocast shown in **Fig. 36D** is shown again as Specimen 1 in **Fig. 37**. It is Falk B, more like other humans in average size, *E* = 1,369.7 ml; and assuming *P* = 70 kg; *EQ* = 6.72. It is comparable to MRI data from the living humans. Endocast length = 16.3 cm; surface area = 682.38 cm^2^; neocortex = 530.32 cm^2^; and neocorticalization = 77.72%.

The Sylvian fissure is a prominent feature of lateral views of human brains, although it is almost never completely evident in endocasts. The endocasts in **Fig. 36C and D** emphasize the inadequate image of the Sylvian Fissure. The difference in volume of more than 300 g amounts to about two standard deviations in living human brains. The size of the smaller endocast is unusual, but its shape in the Sylvian region is like that of other human endocasts.

Only lateral images of primate endocasts are shown in **Fig. 37**. The size differences between them are indicated in the paragraph on each specimen, including left-to-right length and endocast or brain volume *E*; grams and milliliters are equivalent. I note finally that the brain images of chimpanzee and rhesus monkey in **Fig. 37** are of somewhat shrunken specimens prepared from preserved brains used in electrophysiological studies on the right hemispheres. The olfactory bulb areas are small enough in all of these primate specimens to be disregarded, but when present they are included in the left-to-right length measurements.

I refer to the specimens in **Fig. 37** by number and with museum numbers if available. The two braincasts in **Fig. 37** are of the chimpanzee (Fig. 37C) and of the rhesus monkey (Specimen 7). The other “brains” in **Fig. 37** are all endocasts, including the chimpanzee endocast (Specimen 2).

My chimpanzee brain scan was prepared from the University of Wisconsin brain collection (63-397), one of many sent to me by Professor Wally Welker. The chimpanzee endocast was prepared at the University of Witwatersrand and sent to me by Dr. Kristian Carlson, Institute for Human Evolution, University of Witwatersrand. I believe it was prepared for him at the Peabody Museum at Yale from a MCZ Mammals specimen (number unknown), a less adequate preparation from CT scans. Their problem was to combine successive 2D scans into a 3D image, and their program did not completely hide the rings of successive 2D scans. The program used here was more successful, as are other recent programs for rendering 2D scan images into 3D digitized images.

Specimen 7 of **Fig. 37** is the braincast (not endocast) of a rhesus monkey, *Macaca mulatta,* Specimen 62-133 of the Wisconsin brain collection. In contrast to the endocasts, the Sylvian fissure is prominent. Although some gyri and sulci are evident in all of the primate endocasts, they are less marked and certainly less complete than in the brains. One morphological feature in the human brain related to the localization of language is the bilateral asymmetry of the Sylvian, which is somewhat longer on average in the left hemisphere. This asymmetry is not measurable in endocasts.

*Pan troglodytes*

On chimpanzee brain and body size I often use Bauchot & Stephan (1969) for data (e.g., in Jerison, 1979). For this monograph, I scanned the two specimens illustrated in **Fig. 37**. Fig. 37B is the CT-based endocast of an unknown MCZ Mammals mentioned earlier. Fig. 37C is the scan of a chimpanzee brain from the Wisconsin brain collection, which is now housed in the National Museum of Health and Medicine (NMHM Vertebrates WISC 63-307). This braincast is from a presumably shrunken brain: *E* = 307.39 ml; length = 10.1 cm. Assuming a body size *P* = 40 kg; *EQ* = 2.19; surface area = 331.52 cm^2^; neocortex = 267.73 cm^2^; and neocorticalization = 80.76%. Crile & Quiring (1940) reported on a chimpanzee, body size *P* = 52 kg; brain size *E* = 440 g; *EQ* = 2.70. Van Dongen reported a 46 kg body and 405 g brain*; EQ* = 2.63.

For the MCZ chimpanzee endocast: *E* = 371.18 ml; *P* = 50 kg; *EQ* = 2.28; surface area = 278.46 cm^2^; remainder (less olfactory bulbs) = 276.66 cm^2^; and neocorticalization = 71.05%.

*Colobus guereza*

*Colobus guereza* (AMNH Mammals 52217, **Fig. 37** Specimen 4) is an African colobus monkey. The species was listed by Van Dongen (1998) as *P* = 10.5 kg; *E* = 85.27 ml; *EQ* = 1.48. I accept his colobus body weight for my Specimen 4, with the measured length = 7 cm; surface area = 112 cm^2^; neocortex = 76 cm^2^; and neocorticalization = 68.15%.

*Erythrocebus patas*

In this Ethiopian red guenon endocast (AMNH Mammals 52574, **Fig. 37** Specimen 5), *E* = 90.06 ml. Van Dongen reported a body size *P* = 17 kg; *EQ* = 1.14; endocast length = 7.1 cm; surface area = 116.93 cm^2^; neocortex = 91.23 cm^2^; and neocorticalization = 78.02%.

*Hylobates lar*

I recorded the endocast as Number 386 from Falk (**Fig. 37** Specimen 6). In most instances, the number would refer to endocasts given to me by Professor Len Radinsky, but in this case my incomplete notes place it in the Falk collection. Professor Fleagle did not comment on it, nor did he give it a museum number. The measurements would be appropriate for the gibbons. Endocast *E* = 99.32 ml; length = 7.1 cm; *P* (after Nowak, 1999) = 8 kg; *EQ* = 2.07; surface area = 123.4 cm^2^; neocortex = 78.11 cm^2^; and neocorticalization = 63.3%.

*Macaca mulatta*

I have only incomplete data on this rhesus monkey braincast that was given to me by Wally Welker. It is one of his braincasts prepared after neurophysiological experiments (WISC 69-307). It is shown in **Fig. 37** Specimen 7; *E* = 71.61 ml. Having worked with monkeys years ago, I recognized this as small for a laboratory rhesus monkey and guess that it shrank following laboratory fixation and preservation. I recorded 6 kg as its body size based on my experience with such specimens, but I have no additional record of its source. *EQ* = 1.81; length = 6.2 cm; surface area = 114.72 cm^2^; neocortex = 79.45 cm^2^; and neocorticalization = 69.26%.

*Nasalis larvatus*

This nasalis monkey (MCZ Mammals 37328, **Fig. 37** Specimen 8) was from Borneo. I take *E* = 97 ml; *P* = 14 kg; *EQ* = 1.39; length = 5.9 cm; surface area = 121.94 cm^2^; neocortex = 89.15 cm^2^; and neocorticalization = 73.11%.

*Pithecia monachus*

Endocast volume of this New World saki (AMNH Mammals 75981, **Fig. 37** Specimen 9) *E* = 39.73 ml; *P* = 1.5 kg; *EQ* = 2.52; length = 5.9 cm; surface area = 68.05 cm^2^; neocortex = 53.09 cm^2^; and neocorticalization = 78.02%.

*Presbytis johnii*

The volume of the endocast (AMNH Mammals 54644, **Fig. 37** Specimen 10) of this langur (leaf monkey) *E* = 85.85 ml; length = 7.2 cm; *P* = 13.4 kg; *EQ* = 1.27; surface area = 114.26 cm^2^; neocortex = 82.62 cm^2^; and neocorticalization = 72.31%.

*Rhinopithecus (Pygathrix) avunculis*

The endocast (MCZ Mammals 13681, **Fig. 37** Specimen 11) of this snub-nosed langur *E* = 114.21 ml; *P* = 8 kg; *EQ* = 2.38; length = 7.4 cm; surface area = 136.64 cm^2^; neocortex = 99.75 cm^2^; and neocorticalization = 73%.

*Pygathrix nigripes*

The endocast (AMNH Mammals 69555, **Fig. 37** Specimen 12) of this douc langur *E* = 77.71 ml; *P* = 7.5 kg; *EQ* = 1.69; length = 6.5 cm; surface area = 106.08 cm^2^; neocortex = 80.39 cm^2^; and neocorticalization = 75.78%.

*Simias concolor*

The endocast (AMNH Mammals 103359, **Fig. 37** Specimen 13) of this pig-tailed langur *E* = 54 ml; *P* = 7 kg; *EQ* = 1.23; length = 5.8 cm; surface area = 82.54 cm^2^; neocortex = 61.19 cm^2^; and neocorticalization = 74.13%.

*Theropithecus gelada*

The volume of the endocast (FMNH Mammals 8174, **Fig. 37** Specimen 14) of this gelada baboon was *E* = 131.08 ml; *P* = 17 kg; *EQ* = 1.65; length = 8.1 cm; surface area = 146.82 cm^2^; neocortex = 108.74 cm^2^; and neocorticalization = 74.06%.

*Cercocebus (Lophocebus) albigena*

Endocast volume of this gray-cheeked langur (AMNH 52583, **Fig. 37** Specimen 15) *E* = 79.64 ml; *P* = 7.9 kg; *EQ* = 1.67; length = 6.7 cm; surface area = 107.84 cm^2^; neocortex = 86.65 cm^2^; and neocorticalization = 80.35%.

*Cercopithecus pygenthus*

The endocast is **Fig. 37** Specimen 16 and is Fleagle’s Male AM 52468 at AMNH. I assumed the body weight of this guenon to be *P* = 4.2 kg from Nowak (1999); *E* = 71.86 ml; *EQ* = 2.3; length = 6.8 cm; surface area = 101.93 cm^2^; neocortex = 79.91 cm^2^; and neocorticalization = 78.4%.

# References

Argot C. Functional adaptations of the postcranial skeleton of two Miocene boryaenoids (Mammalia, Metatheria), *Borhyaena* and *Prothylacinus*, from South America. Paleontology. 2003; 46: 213–267.

Argot C. Postcranial analysis of a carnivoran-like archaic ungulate: The case of *Arctocyon primaevus* (Arctocyonidae, Mammalia) from the late Paleocene of France. Journal of Mammalian Evolution. 2013; 20: 83–114.

Bauchot R, Stephan H. Encephalisation et niveau évolutif chez les Simiens. Mammalia. 1969; 33: 225–275.

Berta A, Ray CE. Skeletal morphology and locomotor capabilities of the archaic pinniped *Enaliarctos mealsi*. Journal of Vertebrate Paleontology. 1990. 10:141–157.

Cassini GH, Flores DA, Vizcaíno SF. Postnatal ontogenetic scaling of Nesodontine (Notoungulata, Toxodontidae) cranial morphology. Acta Zoologica (Stockholm). 2012; 93: 249–259.

Christiansen P. What size were *Arctodus simus* and *Ursus spelaeus* (Carnivora: Ursidae)? Annales Zoologica Finneci. 1999; 36: 93–102.

Cook HJ. A remarkable new mammal from the lower Chadron of Nebraska. American Midland Naturalist. 1954; 52: 388–391.

Cope ED. The vertebrata of the tertiary formations of the west. Report of the United States Geological Survey of the Territories. Book 1. Washington DC: US Government Printing Office; 1883.

Crile GW, Quiring DP. A record of the body weight and certain organ weights of 3690 animals. Ohio Journal of Science. 1940; 40: 219–259.

Croft DA, Anderson LC. Locomotion in the Extinct Notoungulate *Protypotherium*. Palaeontologia Electronica. March 2008; 11.1.1A. Available from: https://palaeo-electronica.org/2008_1/toc.htm.

Cuvier G. Recherches sur les Ossemens Fossiles, 4th ed. vol. 5 and atlas. Paris: D'Ocagne; 1835.

Damuth J, MacFadden BJ, editors. Body Size in Mammalian Paleobiology: Estimation and Biological Implications. Cambridge and New York: Cambridge University Press; 1990.

Dart RA. *Australopithecus africanus*: the man-ape of South Africa. Nature. 1925; 115: 195–199.

Dechaseaux C. Encéphales de Zénarthes fossils. In: Piveteau J. Traité de paléontologie, tome VI, vol. 2. Paris: Masson; 1958. pp. 636–640.

Dozo MT. The endocranial cast of an early miocene edentate *Hapalops indifferens* Ameghino (Mammalia, Edentata, Tardigrada, Megatheriidae). Comparative Study with Brains of Recent Sloths. Journal für Hirnforschung. 1987; 28(4): 397–406.

Dozo MT, Martínez G. First digital cranial endocasts of late oligocene Notohippidae (Notoungulata): implications for endemic South American ungulates brain evolution. Journal of Mammalian Evolution. 2016; 23: 1–16.

Dubois E. The brain-cast of *Pithecanthropus erectus*. Proceedings of the Fourth International Congress of Zoology; 1898. pp.79–96.

Edinger T. Über Gehirne tertiärer Sirenia Ägyptens und Mitteleuropas sowie der reznten Seekühe. Abhandlung bayerische Akademie Wissenschaften, Mathematische – naturwischenfaten. Abt. N.F. 1933; 20: 5–36.

Edinger T. 1948. *Evolution of the horse brain*: Geological Society of America.

Edinger T. Midbrain exposure and overlap in mammals. American Zoologist. 1964; 4: 5–19.

Edinger T. Paleoneurology 1804-1966: an annotated bibliography. Advances in Anatomy, Embryology and Cell Biology. Berlin: Springer-Verlag; 1975; 49: 12–258.

Emry RJ. The edenulous skull of the North American pangolin, *Patriomanis americanus*. Bulletin of the American Museum of Natural History. 2004; 285: 130-138.

Erfurt J, Métais G. Endemic European artiodactyls. In: Prothero DR, Foss SE, editors. The evolution of the Artiodactyls. Baltimore: Johns Hopkins University Press; 2007. pp. 59–84.

Falk D. The natural endocast of Taung (*Australopithecus africanus*): insights from the unpublished papers of Raymond Arthur Dart. Yearbook of Physical Anthropology. 2009; 52: 49–65.

Fleagle JG. Primate adaptation and evolution. New York: Academic Press; 1999.

Gazin CL. A review of the middle and upper Eocene primates of North America. Smithsonian Miscellaneous Collections. 1958; 136, no. 1.

Gazin CL. A study of the early Tertiary condylarthran mammal *Meniscotherium*. Smithsonian Miscellaneous Collections. 1965; 149, no. 2.

Gidley JW. American Wild Horses. The Scientific Monthly 1927; 25:265-271.

Gingerich PD. Body weight and relative brain size (encephalization) in Eocene Archaeoceti (Cetacea). Journal of Mammal Evolution. 2016; 23: 17–31.

Gunnell GF, Bown TM, Bloch JI, Boyer DM, Janis CM. Proteutheria. Evolution of Tertiary mammals of North America, vol. 2: small mammals, xenarthrans and marine mammals. Cambridge University Press; 2007. pp. 63–81.

Gunnell GF, Rose KD, Rasmussen DT. Euprimates. In: Janis CM, Gunnell GF, Uhen MD, editors. Evolution of Tertiary mammals of North America, vol. 2: small mammals, xenarthrans and marine mammals. Cambridge University Press; 2007. pp. 239–262.

Holbrook LT, Lucas SG, Emry RJ. Skulls of the Eocene perissodactyls (Mammalia) *Homogalax* and *Isectolophus*. Journal of Vertebrate Paleontology. 2004; 24: 951–956.

Holloway RL, Broadfield DC, Yuan MS, Schwartz JH, Tattersall I. The human fossil record, vol. 3: brain endocasts––the paleoneurological evidence. New York: Wiley; 2004.

Hooker JJ. The beginning of the equoid radiation. Zoological Journal of the Linnaean Society. 1994; 112: 29–63.

Hulbert RC Jr. Late Miocene *nannipus* (mammalia: perissodactyla) from Florida, with a description of the smallest hipparionine horse. Journal of Vertebrate Paleontology. 1993; 13: 350–366.

Hunt RM Jr. Small Oligocene amphicyonids from North America (Paradaphoenus, Mammalia, Carnivora). American Museum Novitates. 2001; No. 3331.

Jerison HJ. Evolution of the brain and intelligence. New York: Academic Press; 1973.

Jerison HJ. The evolution of diversity in brain size. In: Hahn ME, Jensen C, Dudek BC, editors. Development and evolution of brain size: Behavioral implications. New York: Academic Press; 1979. pp. 29–57.

Jerison HJ. Brain size and the evolution of mind. The 59th James Arthur Lecture on the evolution of the human brain. New York: American Museum of Natural History; 1991.

Jerison HJ. The study of primate brain evolution: Where do we go from here? In: Falk D, Gibson K, editors. Evolutionary anatomy of the primate cerebral cortex. Cambridge, England: Cambridge University Press; 2001a. pp. 305–337.

Johnson JI. Comparative development of somatic sensory cortex. In: Jones EG, Peters A, editors. Cerebral Cortex, vol. 8B. New York: Plenum Press; 1990. pp. 335–449.

Kelly TS. New Miocene horses from the Caliente Formation, Cuyama Valley Badlands, California. Contributions in Science, Natural History Museum of Los Angeles County. 1995; 455:1–33.

Kihm AJ, Hartman JH. A Reevaluation of the Biochronology of the Brisbane and Judson Local Faunas (late Paleocene) of North Dakota. Bulletin of Carnegie Museum of Natural History. 2004, 36: 97–107.

Lange B. *Mustelictis piveteaui*, mustélidé nouveau des Phosphorites du Quercy. Masson; Annales de Paléontologie. 1970; 3–16.

Legendre S, Roth C. Correlation of carnassial tooth size and body weight in recent carnivores (Mammalia). Historical Biology. 1988; 1: 85–98.

LeGros Clark WE. The Antecedents of Man, 2nd ed. Chicago: Quadrangle Books; 1962.

Loomis FB. The small carnivores of the Miocene. American Journal of Science. 1932; 24: 316–329.

Lyras GA. The evolution of the brain in Canidae (Mammalia: Carnivora). Scripta Geologica. 2009; 139:1–39.

MacFadden BJ. Fossil horses from "eohippus" (*Hyracotherium*) to *Equus*: scaling, Cope's Law, and the evolution of body size. Paleobiology. 1986; 12: 355–369.

MacFadden BJ. Fossil horses: Systematics, paleobiology, and evolution of the family Equidae. Cambridge: Cambridge University Press; 1992.

MacPhee RD. The serrialis bone, interparietals,“X” elements, entotympanics, and the composition of the notoungulate caudal cranium. Bulletin of the American Museum of Natural History. 2014; 384:1–69.

Macrini TE. Description of a digital cranial endocast of *Bathygenys reevesi* (Merycoidodontidae; Oreodontoidea) and implications for apomorphy-based diagnosis of isolated, natural endocasts. Journal of Vertebrate Paleontology. 2009; 29: 1199-1211.

Marsh OC. Small size of the brain in Tertiary mammals. American Journal of Science and the Arts. 1874; 8: 66–67.

Marsh OC. Dinocerata: A monograph of an extinct order of gigantic mammals. Washington: U.S. Geological Survey vol. 10; 1886.

Martin RD. Primate origins and evolution: A phylogenetic reconstruction. London: Chapman & Hall; 1990.

Martínez G, Dozo MT, Gelfo JN, Marani H. Cranial morphology of the Late Oligocene Patagonian Notohippid *Rhynchippus equinus* Ameghino, 1897 (Mammalia, Notoungulata) with emphases in basicranial and auditory region. PLoS ONE. 2016;11(5): e0156558. Available from: <https://doi.org/10.1371/journal.pone.0156558>.

McKenna MC, Bell SK. Classification of mammals above the species level. New York: Columbia University Press; 1997.

Mendoza M, Janis C, Palmqvist P. Estimating body mass of extinct ungulates: a study on the use of multiple regression. Journal of Zoology. 2006; 270: 90–101.

Mihlbachler MC. Species taxonomy, phylogeny, and biogeography of the Brontotheriidae (Mammalia: Perissodactyla). Bulletin of the American Museum of Natural History. 2008; 311:1–475.

Mihlbachler MC, Lucas SG, Emry RJ. The holotype specimen of *Menodus giganteus*, and the “insoluble” problem of Chadronian brontothere taxonomy. New Mexico Museum of Natural History and Science Bulletin. 2004;26:129–35.

Mitchell E, Tedford RH. The Enaliarctinae: a new group of extinct aquatic Carnivora and a consideration of the origin of the Otariidae. Bulletin of the American Museum of Natural History. 1973; 151: 201–284.

Moodie RL. On the endocranial anatomy of some Oligocene and Pleistocene mammals. Journal of Comparative Neurology. 1922; 34: 343–370.

Nowak RM. Walker’s mammals of the world. 2 vols. Baltimore and London: Johns Hopkins University Press; 1999.

Owen R. Description of fossil remains of a mammal (*Hyracotherium leporinum*) and of a bird (*Lithornis vulturinus*) from the London Clay. Transactions of the Geological Society London. 1841; 6: 203–208.

Orliac MJ, Sandrine L, Gingerich PD, Lebrun R, Smith T. Endocranial morphology of Palaeocene *Plesiadapis tricuspidens* and evolution of the early primate brain. Proceedings of the Royal Society B: Biological Sciences. 2014; 281: 20132792.

Osborn HF. The Titanotheres of ancient Wyoming, Dakota, and Nebraska. Washington DC: U. S. Geological Survey, Monograph 55; 1929.

Palmer RW. The brain and brain-case of a fossil ungulate of the genus *Anoplotherium*. Proceedings of the Zoological Society of London. 1913; 83: 878–893.

Patterson B. Some notoungulate braincasts. Field Museum of Natural History Geological Series. 1937; 6: 273–301.

Peterson OA. Report upon the material discovered in the upper Eocene of the Uinta Basin by Earl Douglas in the years 1908-1909, and by OA Peterson in 1912. Annals of the Carnegie Museum. 1919; 12:40–168.

Piveteau J. Traité de paléontologie, tome VI, vol. 2. Paris: Masson; 1958.

Piveteau J. Traité de Paléontologie, tome VI, vol. 1. Paris: Masson; 1961.

Radinsky LB. Origin and early evolution of North American Tapiroidea. Yale University: Bulletin of the Peabody Museum of Natural History 17; 1963.

Radinsky LB. The brain of *Mesonyx*, a Middle Eocene mesonychid condylarth. Fieldiana: Geology. 1976a; 33: 323–337.

Radinsky L. Oldest horse brains: more advanced than previously realized. Science 1976b; 194:626-627.

Radinsky L. Evolution of brain size in carnivores and ungulates. American Naturalist. 1978; 112: 815–831.

Radinsky L. The Fossil Record of Primate Brain Evolution. The James Arthur Lecture. American Museum of Natural History, New York. 1979.

Radinsky L. Brain evolution in extinct South American ungulates. Brain, Behavior and Evolution. 1981; 18: 169–187.

Rothwell T. Phylogenetic systematics of North American *Pseudaelurus* (Carnivora: Felidae). American Museum Novitates. 2003; No. 3403.

Russell DE, Sigogneau D. Étude de moulages endocraniens de mammifères paléocènes. Memoires du Muséum National D’Histoire Naturelle, Paris. 1965; 16: 1–43.

Sanders WJ, Rasmussen DT, Kappelman J. Embrithopoda. In: Werdelin L, Sanders WJ, editors. Cenozoic mammals of Africa. Berkeley: University of California Press; 2010.

Savage RJG, Long MR. Mammal evolution: An illustrated guide. London: British Museum (Natural History); 1986.

Scott WB. A History of Land Mammals in the Western Hemisphere (2^nd^ edition). New York: Macmillan; 1937.

Secord R, Gingerich PD, Smith ME, Clyde WC, Wilf P, Singer BS. Geochronology and mammalian biostratigraphy of middle and upper Paleocene continental strata, Bighorn Basin, Wyoming. American Journal of Science. 2006; 306: 211–245.

Simpson GG. Horses. London and New York: Oxford University Press; 1951.

Stevens MS, Stevens JB. Family Merycoidodontidae. In: Prothero DR, Foss SE, editors. The evolution of Artiodactyls. Baltimore: Johns Hopkins University Press; 2007. pp. 157–168.

Stephan H, Frahm HD, Baron G. New and revised data on volumes of brain structures in insectivores and primates. Folia Primatologica. 1981; 35: 1–29.
Stevens MS, Stevens JB. Merycoidodontinae and Miniochoerinae. The terrestrial Eocene-Oligocene transition in North America. Cambridge University Press, Cambridge. 1996:498–573.

Stuky RR. Eocene bunodont and bunoselenodon Aritiodactyla (“dichobunids”). In: Janis CM, Scott KM, Jacobs L, editors. Evolution of Tertiary mammals of North America. Cambridge: Cambridge University Press; 1998. pp. 358–374.

Thorpe MR. John Day eporeodonts, with descriptions of new genera and species. American Journal of Science, Series 5. 1921; 6: 93–111.

Thorpe MR. The Merycoidodontidae: An extinct group of ruminant mammals. New Haven: Memoirs of the Peabody Museum; Yale University Press; 1937.

Tobias PV. The Brain in Hominid Evolution. New York: Columbia University Press; 1971.

Tomiya S. A new basal caniform (Mammalia: Carnivora) from the middle Eocene of North America and remarks on the phylogeny of early carnivorans. PLOS ONE. 2011; 6:e24146.

Uhen MD. Form, function, and anatomy of Dorudon atrox (Mammalia, Cetacea): An archaeocete from the Middle to Late Eocene of Egypt. University of Michigan Papers on Paleontology. 2004; 34: 1–222.

Van Dongen PAM. Brain size in vertebrates. In: Nieuwenhuys R, ten Donkelaar HJ, Nicholson C, editors. The central nervous system of vertebrates, vol. 3. Berlin: Springer-Verlag; 1998. pp. 2099–2134.

Wall WP. The genus Amynodon and its relationship to other members of the Amynodontidae (Perissodactyla, Rhinocerotoidea). Journal of Paleontology. 1982; 56: 434–443.

Wall WP. Amynodontidae. In Janis C, Scott KM, Jacobs LL, editors. Evolution of Tertiary mammals of North America, vol.1: Terrestrial carnivores, ungulates, and ungulate like mammals. Cambridge University Press, Cambridge, England. 1998. pp. 583–588.

Wang X, Tedford RH, Taylor BE. Phylogenetic systematics of the Borophaginae (Carnivora: Canidae). Bulletin 243. New York: American Museum of Natural History; 1999.

Welker WI. Why does cerebral cortex fissure and fold? A review of determinants of gyri and sulci. In: Jones EG, Peters A, editors. Cerebral Cortex vol. 8B. New York: Plenum Press; 1990. pp. 1–132.

Wells RT, Tedford RH. *Sthenurus* (Macropedia, Marsupopdidae) from the Pleistocene of Lake Callabonna, South Australia. New York: Bulletin of the American Museum of Natural History, No. 225; 1995.

Wesley-Hunt GD, Werdelin L. Basicranial morphology and phylogenetic position of the upper Eocene carnivoramorphan *Quercygale*. Acta Palaeontologica Polonica. 2005;50:837–846.

Wilson JA. Early Tertiary vertebrate faunas, Vieja Group, Trans-Pecos Texas: Agriochoeridae and Merycoidodontidae. Texas Memorial Museum Bulletin. 1971; 18:1–83.

Woodburne MO, editor. Late Cretaceous and Cenozoic Mammals of North America. New York: Columbia University Press. 2004.

Woodward AS. On some remains of *Grypotherium (Neomylodon) listai* and associated mammals from a cavern near Consuelo Cove, Last Hope Inlet, Patagonia. Proceedings of the Zoological Society of London. 1900: 64–79.

Zhou X, Sanders WJ, Gingerich PD. Functional and behavioral implications of vertebral structure in *Pachyaena ossifraga* (Mammalia, Mesonychia). Contributions from the Museum of Paleontology, University of Michigan. 1992; 28: 289–319.
