# Supplementary material for: Digitized endocasts and brains: a perspective on measurements and historical analyses of the evolution of 172 fossil and extant amniote specimens
Source: PeerJ. 2025 Sep 16;13:e19826. doi: 10.7717/peerj.19826 (PMC12447954; doi:10.7717/peerj.19826)
Supplement: Supplemental Information 2 [file peerj-13-19826-s002.docx]

**Additional Information**

**Figure 6. *Titanoides, Arctocyon, Barylambda, Phenacodus*.**

(**C**) *Barylambda schmidti* (FMNH P 26075 and FMNH P 15573). Evidence of neocortex on this endocast is marginal: two small anterior mounds on the frontal surface and a poorly marked olfactory tract; the expanded cerebellar representation in this specimen probably reflects fluid surrounding the cerebellum.

(**D**) *Phenacodus primaevus* (AMNH FM 4369 = FMNH PM 59042). The cast is unusually smooth for a cast prepared from a fossil skull and may have been smoothed to look more brain-like during preparation. Scale bars = 1 cm.

**Figure 7. *Coryphodon, Palaeosyops, Heptodon, Isectolophus* endocasts.**(**D**) Endocast of *Isectolophus latidens* (AMNH FM 12222 = FMNH PM 59179); the odd preservation of its olfactory bulbs and tract are examples of the poor quality of olfactory bulb representation in endocasts, a reason for excluding them from endocast surface area estimates, along with the presence of non-neural tissue.

**Figure 11. *Uintatherium, Menodus* (*Titanotherium*), *Moeritherium, Arsinotherium* endocasts.** (**A**) *Uintatherium* anceps (YPM VP 11036); the anterior portion of the skull was almost certainly drilled out when the skull was prepared for the endocast, and the size of the endocast’s “olfactory bulbs” are grossly overestimated.

(**C**) *Moeritherium* (NHMUK PV M 9176 b); the matrix forms a large mass of material at the ventral border of the olfactory bulbs.

(**D**) *Arsinotherium* *zitelli* (NHMUK PV M 8539); there appears to be matrix added to the right cerebellar area in the region of the flocullus. Convolutions, presumably present in its brain, did not mold the cranial cavity and are not represented in the endocast. It is unique in the sample of land mammals in that the rhinal fissure is not visible, so neocorticalization could not be estimated.

**Figure 12. *Pterodon*, *Cynodictis, Cynohyaenodon*, *Procynodictis* endocasts*.***

(**C**) *Cynohyaenodon cayluxi (*FMNH PM 57153); only its left half was preserved, and although the rhinal fissure was unclear in this image, it was clear enough to estimate neocorticalization. (**D**) *Procynodictis angustidens*, (AMNH FM 95590 = FMNH PM 57168); the rhinal fissure is faintly visible dorsal to the olfactory tract.

**Figure 13. *Cebochoerus, Hylomeryx, Mixtotherium, Chadronia* endocasts*.***

(**C**) *Mixtotherium cuspidatum* **(**FMNH PM 59052); just over half of the endocast could be scanned, and the olfactory bulbs were preserved.

(**D**) *Chadronia margaretae* (AMNH FM 109412 = FMNH PM 57129); endocast is appropriately convoluted for a brain its size, comparable to capybara, and more convoluted than the surprisingly smooth-brained beaver.

**Figure 14. *Anoplotherium, Patriomanis, Poebrotherium, Bathygenys* endocasts*.***

(**A**) *Anoplotherium commune* (NHMUK PV M 3753); endocast edited to remove matrix artifacts from the base of the “medulla”. This endocast is an important example of the railroad-car appearance, elongated to distinguish cerebellum from forebrain as occurs in some Paleogene fossil endocasts.

(**D**) *Bathygenys reevesi* (TMM TXVP 40209-431); the olfactory tract abuts the anterior border of the rhinal fissure, but the fissure continues ventral to neocortex.

**Figure 16. *Merycoidodon, Mesohippus, Promerycochoerus, Hesperocyon* endocasts*.***.

(**D**) *Hesperocyon gregarius* (FMNH PM 58989); sectioned at the midline and the data doubled from the measurements; errors incurred by having only a partial endocast available and, furthermore, the cast of the olfactory bulb and tract is unusually large, adding both to endocast volume and to its total surface area.

**Figure 23. *Adinotherium, Merychippus, Plionictis, Pseudaelurus* endocasts*.***

(**D**) *Pseudaelurus validus* (AMNH FM 61835 = FMNH PM 58867); this species features unusually long olfactory tracts and consequently have enlarged representation of the olfactory system in their brain images.

**Figure 29. *Platygonus, Sthenurus, Thylacoleo, Archaeolemur* endocasts*.***

(**A**) *Platygonus compressus.* (CM VP 12888 = FMNH PM 59058); only half of the endocast was preserved, but it was a relatively clean half, easy to double to estimate its measurements.

(**C**) *Thylacoleo carniflex* (SAMA P18681 = FMNH PM 59244); it is worth noting that the characteristic gyri of the felid and canid endocasts are not a feature of this marsupial carnivore. In felids and canids, the ectosylvian gyri are useful maps of the auditory cortex; they are not a feature in the marsupial.

**Figure 34. *Lutra lutra, Lontra canadensis, Procyon* endocast and braincast*, Nasua.***

(**D**) and (**E**) are not endocranial casts; they are of brains and show their external appearance vividly, although sulcal depths are obscured when casting whole brains. It is clear that although these brains are similar, they are not identical, and it is difficult to make localizations.

**Figure 35. *Phascolarctos*, *Macropus, Vombatus, Taxidea.***

(**B**) *Macropus fulginosus* left hemisphere braincast (MSU 64023); braincast measurements are hardly comparable to those on the endocast because so much midbrain surface is measurable.

**Figure 36. *Chiropotes*, Mandrill, *Homo*-Falk A, *Homo*-Falk B.** Four primate endocasts.

(**D**) *Homo sapiens* (Falk B); the ventrolateral view exposes more of the rhinal fissure, though it is not easy to trace it in this figure; the fissure is often hidden in more familiar lateral views in primates.

**Figure 37. Primate left hemisphere endocasts and braincasts**

The brain images of chimpanzee and rhesus monkey are of somewhat shrunken specimens prepared from preserved brains used in electrophysiological studies on the right hemispheres. The olfactory bulb areas are small enough in all of these primate specimens to be disregarded.
